# From Continuous Observations to Symbolic Concepts: A Discrimination-Based Strategy for Grounded Concept Learning

**DOI:** 10.3389/frobt.2020.00084

**Published:** 2020-06-26

**Authors:** Jens Nevens, Paul Van Eecke, Katrien Beuls

**Affiliations:** Artificial Intelligence Laboratory, Vrije Universiteit Brussel, Brussels, Belgium

**Keywords:** grounded concept learning, language games, hybrid AI, CLEVR, emergent communication

## Abstract

Autonomous agents perceive the world through streams of continuous sensori-motor data. Yet, in order to reason and communicate about their environment, agents need to be able to distill meaningful concepts from their raw observations. Most current approaches that bridge between the continuous and symbolic domain are using deep learning techniques. While these approaches often achieve high levels of accuracy, they rely on large amounts of training data, and the resulting models lack transparency, generality, and adaptivity. In this paper, we introduce a novel methodology for grounded concept learning. In a tutor-learner scenario, the method allows an agent to construct a conceptual system in which meaningful concepts are formed by discriminative combinations of prototypical values on human-interpretable feature channels. We evaluate our approach on the CLEVR dataset, using features that are either simulated or extracted using computer vision techniques. Through a range of experiments, we show that our method allows for incremental learning, needs few data points, and that the resulting concepts are general enough to be applied to previously unseen objects and can be combined compositionally. These properties make the approach well-suited to be used in robotic agents as the module that maps from continuous sensory input to grounded, symbolic concepts that can then be used for higher-level reasoning tasks.

## 1. Introduction

A concept can be described as a mapping between a symbolic label and a collection of attributes that can be used to distinguish exemplars from non-exemplars of various categories (Bruner et al., 1956). In the context of grounded, autonomous agents, these attributes correspond to streams of continuous-valued data, obtained through the agent's various sensors. In order to communicate and reason about the world, agents require a repertoire of concepts that abstracts away from the sensori-motor level. Without this layer of abstraction, communication would happen by directly transmitting numerical observations. Such a system easily leads to errors in communication, for example when the agents observe the world from different perspectives, or when calibration is difficult because of changing lighting conditions or other external factors. To obtain a repertoire of concepts, i.e., mappings from labels to attribute combinations, autonomous agents face two learning problems simultaneously. First, the agents need to find out which attributes are important for each concept. This requires a mechanism for identifying meaningful combinations of attributes from their sensori-motor data streams and attaching a symbolic label to each of these combinations. Second, the agents must be able to recognize instances of particular concepts and distinguish concepts from each other. For representing concepts, we make use of prototype theory (Rosch, 1973), although also other approaches have been proposed in the psychological literature (McCarthy and Warrington, 1990; Squire and Knowlton, 1995; Patalano et al., 2001; Grossman et al., 2002).

A range of different approaches have been applied to concept learning, including version spaces and deep learning techniques. However, we identify a number of drawbacks in these approaches. In version space learning, a concept is represented as an area in a hypothesis space. This space can for example denote the possible ranges of values of various attributes of the concept. Each concept is bound by the most general and the most specific consistent hypothesis. Using positive and negative examples, these boundaries can be updated using the candidate elimination algorithm (Mitchell, 1982). A well-known caveat of this technique, however, is its inability to handle noisy data. In deep learning approaches, concepts are often represented through embeddings, i.e., high-dimensional numerical representations, which lack human-interpretability (see e.g., Mao et al., 2019; Shi et al., 2019). Additionally, as these embeddings are learned in a statistical way, they often fail to adapt to unseen scenarios and require huge amounts of training data. Neither of these approaches offer a learning mechanism that would be suitable for an autonomous agent, i.e., responsive to changes in the environment, able to support incremental learning, and able to dynamically expand the agent's repertoire of concepts.

In this paper, we propose a novel approach to grounded concept learning. Using the language game methodology (Steels, 2001), we set up series of scripted, task-oriented communicative interactions in a tutor-learner scenario. The environment in which these interactions take place is adopted from the CLEVR dataset (Johnson et al., 2017). This environment consists of scenes made up of geometrical objects, where the objects differ in color, size, shape, material, and spatial position. Through the communicative task, an agent must learn the concepts present in this dataset, such as small, red, or left. Learning these concepts requires not only finding relevant attribute combinations (e.g., “r,” “g,” and “b” for color), but also their prototypical values (e.g., “r:44,” “g:76,” and “b:215” for blue). Both the tutor and the learner agent make use of the notion of discrimination, i.e., maximally separating one particular object from the other objects in the scene. Discrimination is an often-used mechanism in experiments on the emergence and evolution of language (Steels, 1997; Vogt, 2002; Pauw and Hilferty, 2012; Wellens, 2012; Bleys, 2016). In language production, the tutor looks for the concept that is maximally discriminating for a particular object, thereby helping the learner to solve the communicative task. The learner, on the other hand, uses the tutor's feedback and discrimination to update its repertoire of concepts after every interaction. This ensures that the concepts are optimally relevant for the communicative task and the environment in which they occur.

The main contribution of this paper is a novel method to represent and learn symbolic concepts that provide an abstraction layer over continuous-valued observations. This method builds on earlier work by Wellens (2012) and extends the discrimination-based learning of concepts represented by weighted combinations of attributes, so that they can be learned from continuous streams of data. Through various experiments, we demonstrate how the learner acquires a set of human-interpretable concepts in a way that is (i) general, (ii) adaptive to the environment, (iii) requires few interactions, and (iv) allows for compositionality.

The remainder of this paper is structured as follows. In section 2, we discuss existing approaches to concept learning. Section 3 introduces the environment in which the agents operate and the language game setup. In section 4, we introduce the experiments, each showcasing a desirable property of our approach. The experimental results are provided and discussed in section 5. Finally, in section 6, we summarize and conclude.

## 2. Related Work

### 2.1. Version Space Learning

One method for representing and learning concepts is through version spaces (Mitchell, 1982). In this method, a concept is represented as an area in a space with dimensionality equal to the number of attributes. The concept area is bounded by both the most specific consistent hypothesis and the most general consistent hypothesis. A hypothesis consists of a combination of attribute values and it is considered consistent when it agrees with the observed examples. With this representation, the simplest way of learning concepts is through the candidate elimination algorithm. Provided with both positive and negative training examples, the algorithm works as follows. The most general and most specific hypotheses are being updated in such a way that the former covers all positive training examples, including as much as possible of the remaining attribute space but excluding any negative examples, and the latter covers all positive training examples with as little as possible of the remaining attribute space. These updates happen in an incremental manner, looking for the minimal specialization for the most general hypothesis and the minimal generalization for the most specific hypothesis.

A major drawback of the candidate elimination algorithm is its inability to handle noisy data. Noisy or wrongly labeled training examples can incorrectly update one or both of the boundaries and recovering from such errors is often difficult. On the positive side, because of the relatively simple representation and learning algorithm, concepts learned using version spaces are often human-explainable and transparent. Furthermore, when the boundaries are allowed to be updated after training, the concepts remain adaptive over time.

### 2.2. Neural Approaches

More recent approaches to concept learning are dominated by deep learning techniques. State-of-the-art results have been achieved by Higgins et al. (2016) and Shi et al. (2019). These two approaches vary strongly in the neural network architecture, the learning regime (e.g., binary or multi-class classification or unsupervised learning), the concept representation (e.g., a label in a classifier or a group of latent variables) and the task or domain in which concepts are being learned (e.g., hand-written characters or generated graphics). However, the aforementioned papers are particularly interesting since both of them take inspiration from human concept learning and incorporate this in their models. For example, how humans require only one or a few examples to acquire a concept is incorporated through one-shot or few-shot learning or how known concepts can be used to recognize new exemplars is achieved through incremental learning and memory modules. Many more approaches to concept learning using deep learning techniques exist (e.g., Wang et al., 2015; Dolgikh, 2018; Xu et al., 2018; Rodriguez et al., 2019). In general, these approaches yield high levels of accuracy but require huge amounts of training data and/or training time. Additionally, the concepts are represented in a way that is often not human-interpretable and the set of concepts is often predefined and fixed over time. Some of the aforementioned approaches tackle one or two of these issues, but not all together.

In other approaches, concepts are learned as a “side effect” while tackling another, typically larger task. In the work by Mao et al. (2019) and Han et al. (2019), not only concepts but also words and semantic parses of sentences are learned in the context of a Visual Question Answering task. Specifically, a perception module learns visual concepts, represented as embeddings, based on the linguistic description of the object being referred to. As reported by Mao et al. (2019), the concepts are acquired with near perfect accuracy (99.9%) and a relatively small amount of training data (5K images), but the resulting concept representations are not human-interpretable. The proposed model does allow for incremental learning and generalizes well to unseen combinations of attributes. This generalization, however, requires fine-tuning the model on a held-out dataset.

### 2.3. The Omniglot Challenge and Bayesian Program Learning

One particular line of research that focusses exclusively on human-like concept learning is centered around the Omniglot dataset (Lake et al., 2015). This is a dataset of hand-written characters from 50 different alphabets. Each character is written by 20 people and stored as both an image and pen stroke data. The Omniglot challenge aims to push forward the state-of-the-art in human-like concept learning. The main challenge consists of a within-alphabet one-shot classification task: given a new character and an alphabet, identify the character in the alphabet that is the same character as the one presented. This task aims to replicate the ability of humans to acquire a new concept with only a single example. Next to this, there are three other tasks designed to test several concept learning-related abilities: parsing of exemplars into parts and relations, generating new exemplars of a given concept and generating new concepts of a particular type.

In his own work, Lake et al. (2015) introduces Bayesian Program Learning (BPL) to tackle the Omniglot challenge. Here, concepts are represented as probabilistic generative models, trained using the pen stroke data and built in a compositional way such that complex concepts can be constructed from (parts of) simpler concepts. In this case, the model builds a library of pen strokes and characters can be generated by combining these pen strokes in many different ways. This approach has many advantages, including the ability to do one-shot learning and a powerful compositional representation of concepts that allows not only to classify concepts but also to generate them. While this model achieves impressive results, learning through pen stroke data offers a limited range of possibilities. Other researchers have tackled the Omniglot challenge, mostly using neural approaches as reported by Lake et al. (2019). Almost all of them have focussed on the one-shot classification task using the image data as input. As a result, the BPL approach remains the SOTA model for all tasks in the Omniglot challenge.

### 2.4. Reinforcement Learning

Concept learning has also been approached from a reinforcement learning perspective. In this context, a concept is regarded as an abstraction over an agent's states or actions. Abstraction over discrete states can be achieved through tile-coding (Sutton, 1996). Recently however, following advances in the domain of deep reinforcement learning, abstraction over continuous states is often performed through function approximation (Mnih et al., 2015). Abstraction over actions is commonly achieved through the use of options (Sutton et al., 1999).

One line of research that is particularly relevant to our approach is the work by Konidaris and colleagues. Initially, the authors mapped propositional symbols to a set of low-level states (Konidaris et al., 2014). These states were obtained from the continuous environment through a classifier. A planning problem is then solved using the propositional symbols as operators, which can be translated to sets of low-level states, executed in the environment. In later work, the set-based representation was replaced by a probability distribution, to better capture the uncertainty about the successful execution of each high-level step (Konidaris et al., 2015, 2018). Again, this approach was validated through a planning problem in a continuous state space, where policies for high-level planning problems in a game environment, such as “obtain key” or “obtain treasure,” could be computed efficiently.

The symbolic high-level steps can be represented in a human-interpretable way, as the pre- and postconditions can be easily visualized in the game environment. Additionally, the model proposed by Konidaris et al. (2015) can be learned efficiently with relatively few data points: 40 iterations of 100 randomly chosen actions were used to extract the high-level steps. As is typical in a Reinforcement Learning setting, the planning steps are learned through experience. Hence, new planning steps must be learned by collecting new experiences specific to this concept. Additionally, the resulting steps are relatively domain-specific. No experiments are reported that investigate generality, e.g., would jump-left generalize to other game settings, or adaptivity, e.g., does the concept jump-left change when the game physics change.

### 2.5. Robotics

A large body of work exists in the robotics community that considers various tasks very similar to what we refer to as concept learning. Common names for this task include symbol emergence, perceptual anchoring, affordance learning, and category learning.

As a first approach, we consider the task of perceptual anchoring. The goal of perceptual anchoring is to establish and maintain a link between symbols and sensor data that refer to the same physical object (Coradeschi and Saffiotti, 2003). This link should remain stable through time and space, e.g., when an object moves through a robot's view, when it is covered by another object, or when it disappears and later reappears. The symbol system can manipulate individual symbols, referring to objects as a whole, but also predicates reflecting properties of the objects. Different representations can be used by the sensor system, e.g., a set of continuous-valued features or a vector in some embedding space. An anchoring system can be bottom-up, starting from the perceptual level, and top-down, starting from the symbolic level. In the context of perceptual anchoring, the combination of a symbol, a set of predicates and sensor data can be considered a single concept.

In recent work, a bottom-up perceptual anchoring system was combined with a probabilistic symbolic reasoning system (Persson et al., 2019). This approach allowed to improve the overall anchoring process by predicting, on the symbolic level, the state of objects that are not directly perceived. There are multiple advantages to this approach. First, the authors achieve high accuracy (96.4%) on anchoring objects and maintaining these anchors in dynamic scenes with occlusions, using relatively little training data (5400 scenes, 70% used for training). Additionally, their system is completely open-ended and allows for incremental learning, since the anchor matching function will simply create new anchors when it encounters previously unseen objects. The anchor matching function, in some way a similarity measure, is closely related to the notion of discrimination. The difference being that discrimination also takes the other objects into account. Finally, the representation of a concept can be human-interpretable, depending on the representation of objects in the sensor system and the corresponding symbols and predicates.

For a second approach, we focus on affordance learning. With this approach, the focus lies on the interaction between the perceptual system and the motor system of an autonomous agent. Put differently, an affordance can be considered as a learned relation between an action in the environment, caused by the motor system, and the effect observed in the environment, captured by the perceptual system (Şahin et al., 2007). Building on this, the agent can learn concepts in terms of affordances. As proposed by Ugur et al. (2011) and further worked out in Ugur and Piater (2015a,b), affordances can be grouped together in effect categories. These are consequently mapped to clustered object properties to form a particular concept. For example, the concept ball is an object with spherical properties that exhibits the roll-effect when pushed and the disappear-effect when lifted, as it rolls off the table when dropped. In these works, the authors use concepts learned through their affordances in plan generation and execution, with an agent being capable of planning the necessary actions involving specific objects to reach a given goal state. This approach offers a more action-centric view on the agent's world, which is complementary to our approach. It not only allows an agent to recognize and describe objects in the world, but also correctly act on them. The concepts that are acquired, combining effect categories with object properties, offer a transparent view. The effect categories are expressed in terms of change in visibility, shape and position, and the object properties are stored in a numerical vector with explainable entries, such as features relating to position and shape (Ugur et al., 2011). Additionally, since the concepts are learned through unsupervised exploration, the proposed model is adaptive to the environment. New concepts can be added incrementally through additional exploration and learned concepts can be progressively updated (Ugur and Piater, 2015b). As is typical in robotics, the proposed approach combines learning in simulation and using physical robots. The concepts, specifically, could be acquired after only 4,000 simulated interactions (Ugur et al., 2011). The robot is used to validate these concepts in several planning problems. Finally, as the agent assesses the object features relevant for each effect category, the resulting mappings offer some generality, e.g., a ball exhibits the same effect categories regardless of its color.

Other approaches take a probabilistic perspective on concept learning, similar to Lake et al. (2015), but focussing on the domain of robotics. Concepts are learned through unsupervised online learning algorithms, combining multi-modal data streams (most often perceptual data and raw speech data) through statistical approaches such as Bayesian generative models or latent semantic analysis (Nakamura et al., 2007; Aoki et al., 2016; Taniguchi et al., 2016, 2017). Through this integration of data streams, the acquired concepts constitute mappings between words and objects, as studied by Nakamura et al. (2007) and Aoki et al. (2016), or between words and spatial locations, as studied by Taniguchi et al. (2016, 2017). The latter further used these concepts to aid a mobile robot in generating a map of the environment without any prior information. The statistical methods have the advantage of being able to infer a considerable amount of information from a limited number of observations, and are therefore suitable for use in robotics scenarios. Additionally, they offer model interpretability to a certain extent, through a graphical model representation such as a Bayesian network. Finally, the proposed models are adaptive to changes in the environment and offer incremental learning through the online learning algorithms.

Among the various approaches to concept learning discussed so far, our proposed approach is most closely related to the robotics literature, as many of these studies deal with similar issues such as grounding, adaptivity, generality, and fast learning. For a more comprehensive overview on symbol emergence from the viewpoint of cognitive systems/robotics, we refer to Taniguchi et al. (2018).

### 2.6. Discrimination-Based Learning

One particular experiment by Wellens (2012) has heavily inspired this work. Wellens makes use of the language game methodology to study multi-dimensionality and compositionality during the emergence of a lexicon in a population of agents. In this language game, called the compositional guessing game, the speaker tries, using language, to draw the attention of the listener to a particular object in a shared scene. Each object in such a scene is observed by the agent as a collection of symbolic attributes, e.g., “a-1,” “a-2,” “a-3” and so on. The words used by the agents have one or multiple of these same symbols as their meaning (multi-dimensionality) and the agents can use multiple words to describe a particular object (compositionality). At the end of a game, the agents give each other feedback on the outcome of the game and the speaker points to the intended object in case of failure. This setup leads to a large amount of uncertainty for the agents, as they should find out what part of the meaning should be linked to which word in the multi-word utterance.

**Figure 1:**
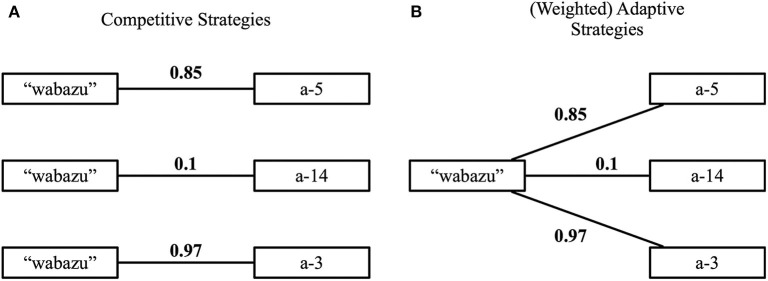
**(A)** Competitive strategies enumerate competing hypotheses. **(B)** Adaptive strategies allow the meaning to be shaped gradually. By adding weights, this can be done in a more fine-grained manner.

In his work, Wellens proposes two distinct types of strategies for reducing this uncertainty: competitive strategies and adaptive strategies. Both make use the notion of discrimination, i.e., maximally separating one object from the others, for both language production (the speaker) and interpretation (the hearer). However, in the former type of strategies, the agents explicitly enumerate competing hypotheses (i.e., the same word with a different meaning) and mechanisms are in place to gradually reduce this enumeration. This soon becomes intractable, leading to scaling issues in environments with many objects or many attributes per object. The latter type of strategies, on the other hand, avoids enumerating competing hypotheses. Instead, only a single meaning, composed of a set of attributes, is kept for each word. Over the course of interactions, this meaning is gradually being shaped based on the feedback provided after each interaction. How this shaping is implemented depends on the particular strategy. Adaptive strategies focus on re-use, allowing agents to use words even when the associated meanings are not (yet) fully compatible with the topic object. Figure 1 illustrates the difference between the two types of strategies.

Within the realm of adaptive strategies, a distinction is made between the baseline adaptive strategy and the weighted adaptive strategy. In the former strategy, the ideas underpinning adaptive strategies are implemented in a rather crude way. The agents gradually shape the meaning of words simply by adding or removing attributes from the set, based on the feedback after the game. The latter strategy offers a more gradual shaping of the meaning. Here, the meaning is no longer a regular set of attributes but instead it is a weighted set. Each attribute receives a score, expressing the certainty that the attribute is important for the word it is linked to. Based on the received feedback, agents cannot only add or remove attributes, but also alter the score of attributes to reflect changes in certainty. Over time, the meanings are shaped to capture attribute combinations that are functionally relevant in the world, driven by the force to obtain communicative success and the notions of discrimination and alignment. For more details about the compositional guessing game and the various strategies, we refer to Wellens (2012).

Our approach to concept learning is heavily inspired by the weighted adaptive strategy. As we will discuss later on, concepts in our approach are also represented by weighted attribute sets. However, where previous work only considers symbolic attributes, we extend this approach to continuous-valued attributes, introducing the need for more sophisticated representations and processing mechanisms.

## 3. Methodology

The goal of this work is for an agent to distill meaningful concepts from a stream of continuous sensory data through a number of communicative interactions called language games. These interactions are set in a tutor-learner scenario and take place in a shared environment consisting of scenes of geometric shapes. Driven by the communicative task and the notion of discrimination, the agent will gradually shape its repertoire of concepts to be functional in its environment. In this section, we elaborate on the language game methodology (section 3.1), the environment in which the agents operate (section 3.2), the concept representation and update mechanism as used by the learner (section 3.3) and the mechanisms used by the tutor (section 3.4).

### 3.1. Language Game

The language game methodology is commonly used to study how a population of agents can self-organize a communication system that is effective and efficient in their native environment. By playing language games, agents take part in a series of scripted and task-oriented communicative interactions. A language game is typically played by two agents from the population, one being the speaker and another being the hearer. There is no central control and the agents have no mind-reading capabilities. The agents are only allowed to communicate through language. After a number of games, the population converges on a shared communication system through selection and self-organization. This methodology has been used to study the emergence of a wide range of linguistic phenomena, including grammatical agreement (Beuls and Steels, 2013), color lexicons (Bleys, 2016), argument marking (Lestrade, 2016), quantifiers (Pauw and Hilferty, 2012), spatial language (Spranger and Steels, 2012), case (van Trijp, 2016), etc.

The language game in this work is set up in a tutor-learner scenario. The tutor is an agent with an established repertoire of concepts, while the learner starts the experiment with an empty repertoire. The tutor is always the speaker and the learner is always the listener. Before each game, both agents observe a randomly sampled scene of geometric shapes. The environment itself will be explained in greater detail in section 3.2. For now, we note that the tutor has access to a high-level symbolic annotation of the scene, while the learner observes the scene through streams of continuous data. The symbolic annotation constitutes the ground-truth of the scene and the learning target for the learner agent. This avoids having to manually design a number of concepts in terms of the observed data stream for the tutor, which could bias the system.

The interaction script, which are the steps both agents go through during a single language game, goes as follows. The tutor starts the interaction by choosing one object from the scene as the topic. Using the symbolic annotation, the tutor looks for a concept that optimally discriminates the topic and utters it. By looking for the most discriminative concept, the tutor is actively trying to help the learner in solving the communicative task. If the topic cannot be discriminated using a single concept, the tutor picks another object or scene. This restriction will be lifted later on in one of the more advanced experiments. The learner receives this word and checks its repertoire of concepts. If the concept denoted by this word is unknown, the learner indicates failure to the tutor. Alternatively, if the learner does know the word, it will try to interpret the corresponding concept in the current scene. In other words, the learner will look for the object that best matches the concept. The learner points to this object and the tutor provides feedback on whether or not this is correct.

**Figure 2:**
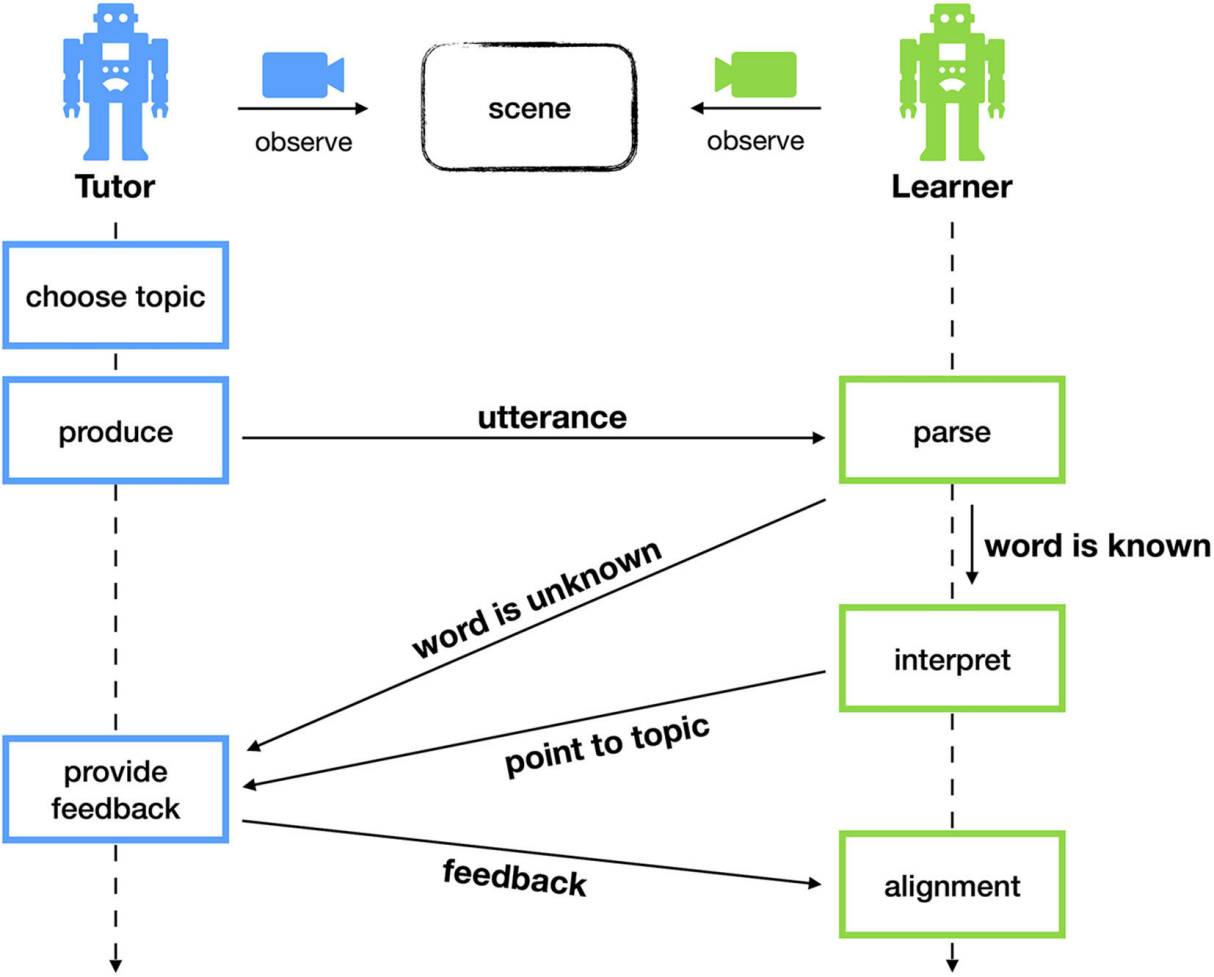
During a single interaction, both agents observe a scene of geometric shapes. The tutor chooses a topic and produces a word denoting a concept that discriminates this topic. The learner looks up this word in his repertoire. If the word is known, the learner tries to interpret this in the scene. Otherwise, the learner indicates failure. After the interaction, the tutor provides feedback to the learner, allowing it to learn.

After each interaction, the tutor provides feedback by pointing to the intended topic. This is a learning opportunity for the learner. We call this phase of the game “alignment.” If the concept was unknown for the learner, it is now able to create a new concept. At this stage, the learner cannot yet know which attributes are important for the concept. It does know, however, that the tutor could discriminate the topic using this concept. Thus, the learner stores an exact copy of the topic object as the initial seed for the corresponding concept. Each attribute receives an initial score of 0.5, reflecting the uncertainty that the attribute is important for the newly created concept. Alternatively, if the learner did know the concept, it can refine its representation using the newly acquired example. This involves updating the prototypical values and the certainty scores of the attributes. We elaborate on this mechanism in section 3.3. A schematic overview of the complete interaction script is shown in Figure 2.

Note that in our description of the interaction script in the previous paragraphs, we have used the words “concept” and “word” interchangeably. We will continue to do so in the remainder of this paper, as in the experiments that we describe, there is a one-to-one correspondence between words and concepts.

To evaluate the learner agent, we measure both communicative success and concept repertoire size. Communicative success indicates whether or not the interaction was successful. In other words, it tells us if the learner could successfully use the concept in interpretation and consequently points to the topic intended by the tutor. Also, we can monitor the number of interactions required to reach a particular level of communicative success, indicating the speed at which the agent is learning. By keeping track of the size of the learners concept repertoire over time, we can check how many interactions are required for the learner to acquire all concepts known by the tutor. In the experimental environment, there are 19 concepts to be learned in total. These are summarized in Table 1.

**Table 1:** All concepts in the experimental environment.

**Shapes**	**Colors**	**Sizes**	**Materials**	**Positions**
CUBE	BLUE	LARGE	METAL	BEHIND
CYLINDER	BROWN	SMALL	RUBBER	FRONT
SPHERE	CYAN			LEFT
	GRAY			RIGHT
	GREEN			
	PURPLE			
	RED			
	YELLOW			

### 3.2. Environment

#### 3.2.1. The CLEVR Dataset

**Figure 3:**
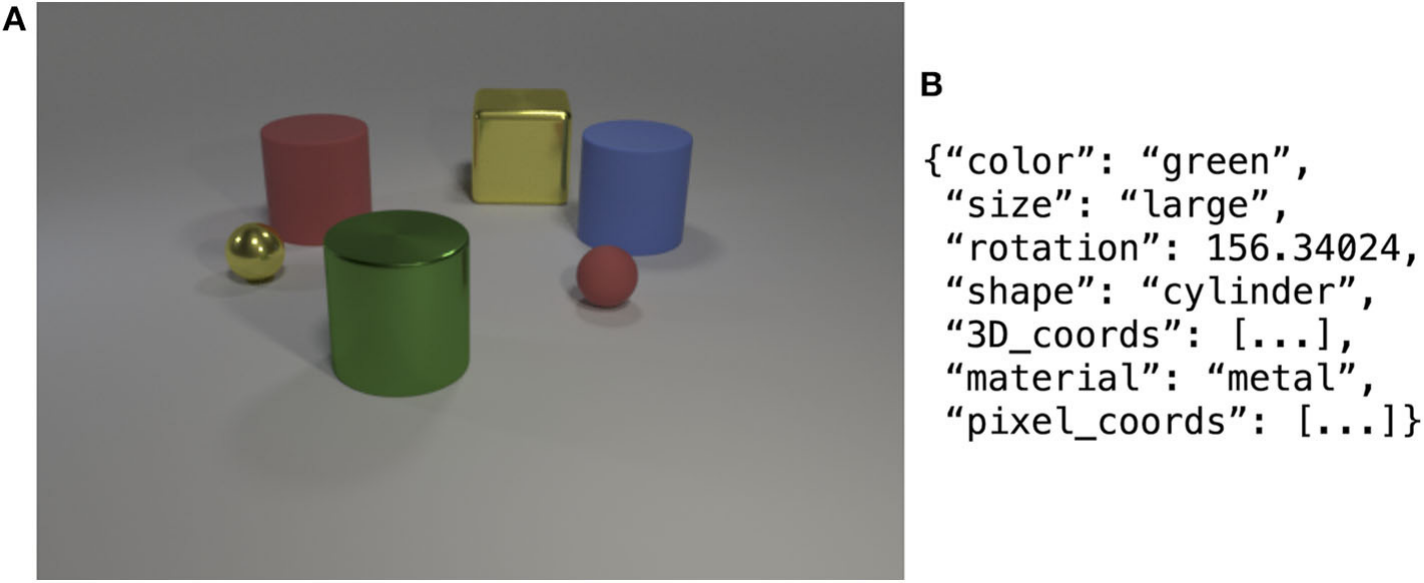
Example image from the CLEVR dataset **(A)** with the corresponding symbolic annotation of a single object **(B)**, namely the green cylinder.

The agent's environment is based on the CLEVR dataset (Johnson et al., 2017). This dataset contains 100K rendered scenes of geometric objects. Each scene contains between 3 and 10 randomly placed objects. The objects have four basic properties: color, size, material, and shape. In total, there are 8 distinct colors, 2 sizes, 2 materials, and 3 shapes. Next to an image of the scene, there is also a ground-truth symbolic annotation, encoded in JSON format. An example scene and annotation are shown in Figure 3. The CLEVR dataset is split into a training set (70K images), a validation set (15K images) and a test set (15K images). In this work, we only make use of the images of the validation set, as no ground-truth annotations are available for the test set. Additionally, since the language game paradigm features online interactive learning, there are no separate training and test phases. The agent is evaluated whilst learning and hence, no held out dataset is required. The CLEVR dataset is ideal for concept learning experiments, as the dataset was specifically designed to avoid dataset biases as much as possible. In practice, this means that across the scenes, there will be as many blue objects as red objects, as many cubes as cylinders, etc.

The learner agent observes its environment through streams of continuous-valued sensor data. To achieve this, the CLEVR scenes need to be transformed into numerical data. We consider two ways of making this transformation. As a first method, we use manually written rules and procedures to transform the symbolic JSON annotation into numerical data. This method is explained in section 3.2.2. For the second method, we use a state-of-the-art Mask R-CNN model (Yi et al., 2018) to detect and segment the objects directly from the image. Section 3.2.3 is dedicated to this method.

#### 3.2.2. Simulated Attributes

The first method starts from the symbolic scene annotations and transforms these into continuous-valued attributes based on simple rules and procedures. We provide an overview of these rules in Table 2. Each symbolic attribute is mapped to one or more continuous attributes with a possible range of values. For example, color is mapped to three attributes, one for each channel of the RGB color space, and size is mapped to a single attribute, namely *area*. We also include the x- and y-coordinates. These attributes were already present in the CLEVR dataset and are simply adopted.

**Table 2:** Rules used to transform symbolic object properties to continuous-valued attributes.

**Symbolic**	**Continuous**	**Values**	**Jitter**
Color	*R*	[0, 255]	±[0, 2]
	*G*	[0, 255]	±[0, 2]
	*B*	[0, 255]	±[0, 2]
Shape	*nr-of-sides*	{1, 3, 6}	/
	*nr-of-corners*	{0, 2, 8}	/
	*Width-height ratio*	[0, 1]	/
Size	*Area*	[0, 100]	±[0, 15]
Material	*Roughness*	[0, 10]	±[0, 2.5]
	*x-coordinate*	[0, 500]	/
	*y-coordinate*	[0, 300]	/

*Note that objects in the CLEVR dataset already have xy-coordinates*.

The values for the various attributes are not chosen arbitrarily. For color concepts, e.g., red, we use the RGB value that was used during the image rendering process of the CLEVR dataset^1^. This value is used as a seed value and random jitter is added. The same technique is used for the size-related concepts. The amount of jitter is shown in the rightmost column of Table 2. Generating the continuous attributes for the shape-related attribute proceeds as follows. We consider a sphere to have 1 side, 0 corners and a width-height ratio of 1, a cylinder to have 3 sides, 2 corners and a width-height ratio of 0.5 and a sphere to have 6 sides, 8 corners and a width-height ratio of 1. Finally, material is identified by a measure of surface roughness.

Obtaining sensory data in this way is straightforward and creates a controlled environment. Indeed, even with the presence of random jitter, there is no overlap between different instances of a particular concept, such as blue and cyan or large and small. For each particular type of concept, every instance takes up a disjoint area in the space of continuous-valued attributes. This makes the concept learning task easier and allows us to validate the proposed learning mechanisms before moving to an environment with more realistic perceptual processing.

#### 3.2.3. Extracted Attributes

To test our approach using more realistic perceptual processing, we make use of a state-of-the-art Mask R-CNN model to detect and segment the objects directly from the image. After segmentation, we extract a number of numerical attributes from the proposed segments. With this approach, different instances of a particular concept will no longer take up disjoint areas in the attribute space. Additionally, the numerical values will be subject to more noise due to variations in the images such as overlapping objects, lighting conditions or shade effects.

For object detection, we use a pre-trained neural network model developed by Yi et al. (2018) using the Mask R-CNN model (He et al., 2017) present in the Detectron framework (Girshick et al., 2018). Given an image, this network generates a mask for each of the objects in the scene. All masks with a certainty score below 0.9 are removed. The model was pre-trained on a separately generated set of CLEVR images. For training regime details, we refer to Yi et al. (2018). To our knowledge, there was no separate evaluation of the object detection accuracy.

We combine the obtained segments with the original image to extract a number of continuous-valued attributes. These are summarized in Table 3. As with the previous environment, we foresee a number of continuous attributes for each symbolic attribute of the CLEVR objects. For colors, we extract both the mean and standard deviation of the color of the region, expressed in the HSV color space and split for each channel. For shapes, we extract the estimated number of corners, the hamming distance between the shape's contour and the enclosing circle, and the width-height ratio. The size-related attributes are straightforward, except for the last two. The *bb-area ratio* expresses the ratio between the area of the region and the area of the rotated bounding box. Similarly, the *image-area ratio* expresses the ratio between the region's area and the area of the entire image. Finally, the material of objects is expressed by the ratio of both dark and bright pixels. These attributes are based on the idea that the metal objects are more reflective and thus contain more bright pixels.

**Table 3:** Mapping from symbolic attributes to continuous attributes obtained by the image segmentation process.

**Symbolic**	**Continuous**	**Values**
Color	*Mean-H*	[0, 255]
	*Mean-S*	[0, 100]
	*Mean-V*	[0, 100]
	*Std-H*	ℝ^+^
	*Std-S*	ℝ^+^
	*Std-V*	ℝ^+^
Shape	*nr-of-corners*	ℝ^+^
	*Hamming distance*	[0, 1]
	*Width-height ratio*	[0, 1]
Size	*Width*	ℝ^+^
	*Height*	ℝ^+^
	*Area*	ℝ^+^
	*Bounding-box area*	ℝ^+^
	*bb-area ratio*	[0, 1]
	*Image-area ratio*	[0, 1]
Material	*Bright-pixels*	[0, 1]
	*Dark-pixels*	[0, 1]
	*Angle*	[0, 180]
	*x-coordinate*	[0, 480]
	*y-coordinate*	[0, 320]

### 3.3. Concept Representation

**Figure 4:**
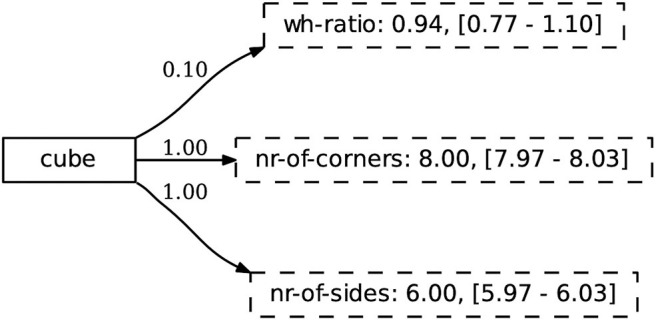
The concept cube is linked to a weighted set of attributes. The weight represents the certainty of an attribute belonging to the concept. Each attribute is modeled as a normal distribution that keeps track of its prototypical value (i.e., the mean) and the standard deviation. The values between square brackets denote two standard deviations from the mean. These are not used in similarity calculations directly, but give an indication of the observed range of prototypical values.

A concept is represented as a mapping from a symbolic label, in this case used as a word, to a set of continuous-valued attributes. Similar to Wellens (2012), we make use of a weighted set representation where each concept-attribute link has a score (∈[0, 1]), representing the certainty that the given attribute is important for the concept. In contrast to Wellens (2012), the attributes are continuous, represented through a normal distribution. This enables the use of such concepts in grounded, embodied scenarios. An example concept is shown in Figure 4.

To computationally operationalize this concept representation in a language game scenario, we require two pieces of functionality: the ability to match a concept to an object and the ability to update an existing concept representation. The former is used by the learner during interpretation, while the latter is used during alignment.

#### 3.3.1. Matching a Concept to an Object

In order to match a concept to an object from the environment, we should foresee some form of distance or similarity measure. Based on this measure, the agent can decide whether or not a particular concept is applicable or discriminative for a particular object, e.g., during interpretation. This idea is similar to Wellens (2012), since it allows an agent to use a concept even if it does not exactly match a particular object. However, Wellens (2012) only considers symbolic attributes, allowing him to implement such a measure using set operations. In this work, we make use of a continuous similarity measure. Specifically, the similarity between a concept *C* and an object *O* can be computed by the average similarity between each of the attributes, weighted by the certainty that an attribute belongs to the concept. Formally, the similarity *S*(*C, O*) is implemented as follows:

(1) $$\begin{array}{l} S\left(C,O\right)=\frac{1}{\left|A_{c}\right|}\underset{a\in A_{C}}{\sum }c\left(C_{a}\right)*S^{\prime }\left(C_{a},O_{a}\right) \end{array}$$

where *A*_*C*_ is the set of attributes linked to concept *C*, |*A*_*c*_| represents the number of attributes, *c*(*C*_*a*_) returns the certainty score for a certain attribute *a* in concept *C* and *C*_*a*_ and *O*_*a*_ represent the attribute value for the attribute *a* in the concept *C* and object *O*, respectively.

Given the above definition of a similarity measure *S* between a concept and an object, we need the similarity measure *S*′ for a particular attribute *a* of the concept and object, respectively. For this, we represent the attribute value within a concept (*C*_*a*_) as a normal distribution. The similarity function *S*′ is based on the *z*-score of the attribute value of the object (*O*_*a*_) with respect to this normal distribution. We embed the *z*-score in a linear function to transform a small *z*-score in a high similarity value and a large *z*-score in a low similarity value. This function maps a *z*-score of 0 to a similarity of 1 and when a *z*-score reaches 2, the similarity has dropped to 0. If the *z*-score would be larger than 4, the similarity is cut off at −1. The similarity measure *S*′ can be expressed with the following equation:

(2) $$\begin{array}{l} S^{\prime }\left(C_{a},O_{a}\right)=\max\left(\frac{\left|-z_{O_{a}}\right|}{2}+1,-1\right) \end{array}$$

where *z*_*O*_*a*__ refers to the *z*-score of the attribute value of the object *O*_*a*_ with respect to the attribute of the concept, *C*_*a*_, represented as a normal distribution.

Given that the similarity function *S*′ returns a value between −1 and 1 and the score is always between 0 and 1, the similarity measure *S* also returns a value between −1 and 1.

#### 3.3.2. Updating Concepts

After each game, the concept used in that game can be updated in terms of both the prototypical value and the certainty score of each attribute. This way, the agent can gradually shape its concept representation to fit the environment, again similar to Wellens (2012). The update mechanism relies on the feedback given by the tutor after the interaction. Specifically, the learner will update the concept it used during the interaction to be closer to or better fit with the topic object. This update procedure works in two steps:

1. The agent updates the prototypical value of all attributes in the concept. Here, we choose to update all attributes since the certainty scores of the attributes might not yet be stable. When a particular attribute suddenly becomes important, e.g., because of changes in the environment, we also want its value to reflect the examples already seen. The update mechanism makes use of Welford's online algorithm (Welford, 1962). This is an online algorithm that specifies recurrence relations for the mean and standard deviation. This allows us to recompute the mean and standard deviation of the distribution by adding a single observation, without the need to store all observations. On the implementation level, each attribute keeps track of the number of observations *N*, the prototypical value *p*_*n*_ and the sum of squared differences from the current mean *M*_2,*n*_ with *n* denoting the current interaction. The latter is initialized at 0.05. Given a new observation *x*_*n*_, these values can be updated using the following equations:
  $$\begin{array}{l} \;N=N+1\\ \;\delta_{1}=x_{n}-p_{n-1}\\ \;p_{n}=p_{n-1}+\frac{\delta_{1}}{N}\\ \;\delta_{2}=x_{n}-p_{n}\\ M_{2,n}=M_{2,n-1}+\left(\delta_{1}*\delta_{2}\right) \end{array}$$
  The standard deviation, required in the similarity calculations discussed above, can be computed from *N* and *M*_2,*n*_ as follows:
  $$\sigma =\sqrt{\frac{M_{2,n}}{N}}$$
2. The agent will increase the certainty of the subset of attributes that is most discriminative for the topic. The certainty score is decreased for all other attributes. A subset of attributes is discriminative when it is more similar to the topic than to any other object in the scene. Since this can be true for multiple subsets, we define the most discriminative subset as the one where the difference between the similarity to the topic and the most similar other object is maximized. Thus, during the update procedure, we not only make use of the topic object itself, but also compare this to other objects in the scene. This ensures that the combination of attributes, and ultimately the entire repertoire of concepts, is functionally relevant in the agent's environment. To compute the most discriminative subset of attributes, we make use of the similarity functions *S* and *S*′ as defined above. Finally, to reduce the computational load, not all subsets of attributes are considered. These are filtered to contain at least the set of attributes that are discriminative on their own. The procedure to update the certainty scores can be summarized as follows:
  - Identify the discriminative attributes, i.e., attributes that are more similar to the topic than to any other object in the scene. Here, we use similarity function *S*′. This yields e.g., *area* and *nr-of-corners*.
  - Compute all subsets of the attributes of the concept.
  - Filter all subsets to contain at least the attributes found in the first step. This yields subsets such as {*area, nr-of-corners*}, {*area, nr-of-corners, wh-ratio*}, {*area, nr-of-corners, roughness*}, etc.
  - Find discriminative subset(s) of attributes, i.e., the subset for which the similarity to the topic is larger than to any other object in the scene. Here, we use similarity function *S*.
  - The previous step can produce multiple subsets. We take the one that maximizes the difference in similarity between the topic and the most similar other object.
  - Increase the certainty score of the attributes in this subset, and decrease the certainty score of all other attributes.

While this concept representation is easy to grasp, there is however an important assumption, namely that the attribute values are modeled using normal distributions. Statistical testing, using the normality test by D'Agostino and Pearson (d'Agostino, 1971; D'Agostino and Pearson, 1973), tells us that this is not the case for any of the attributes. The distributions of the attributes do come close to normal distributions but have thinner tails at both ends. Still, this can be viewed as odd, especially for some of the studied concepts. Take the concept left as an example. It is important to note that the concept of left refers to “left in the image” and not “left of another object.” With this definition of left, the x-coordinate is an important attribute for this concept. If we consider the images of the CLEVR dataset, the x-coordinate of an object can be anywhere between 0 and 480. In this setting, we consider an object to be left when the x-coordinate is smaller than 240. The bulk of objects that can be considered left will not be close to 0, nor close to 240, but somewhere in between, e.g., around x-coordinate 170. From this, it is easy to see that our assumption will not cause many issues in this particular dataset, but in general one could argue that objects with an x-coordinate smaller than 170 can actually be considered “more left,” while objects with an x-coordinates larger than 170 are gradually “less left.” This is currently not captured by our concept representation.

### 3.4. Tutor Behavior

As mentioned in section 3.1, the tutor looks for the smallest set of concepts that discriminates the topic from the other objects in the scene, based on the symbolic ground-truth annotation of the scene. Given a topic that can be described symbolically as (green, cube, large, rubber, left, front), the tutor will try to describe this with a single concept. Traversing the concepts of the topic in a random order, the tutor will check if no other objects in the scene share this concept. For example, if the topic is the only cube in the scene, the concept cube will be returned. In most experiments, we restrict the tutor to only use a single concept to describe an object. In some scenes, however, it is impossible to describe an object with a single, discriminative concept. When this is the case, the tutor will choose a new topic object or sample a new scene.

In the compositional learning experiment, discussed in section 4.4, we lift the single-word restriction. There, if no single discriminative concept can be found, the tutor will try all subsets of two concepts. For example, there might be multiple cubes and multiple green objects, but exactly one green cube. In this case, the combination of green and cube is discriminative. Again, these subsets are considered in a random order. This procedure can be repeated for subsets of three concepts and four concepts, until a discriminative subset is found.

## 4. Experimental Setup

**Figure 5:**
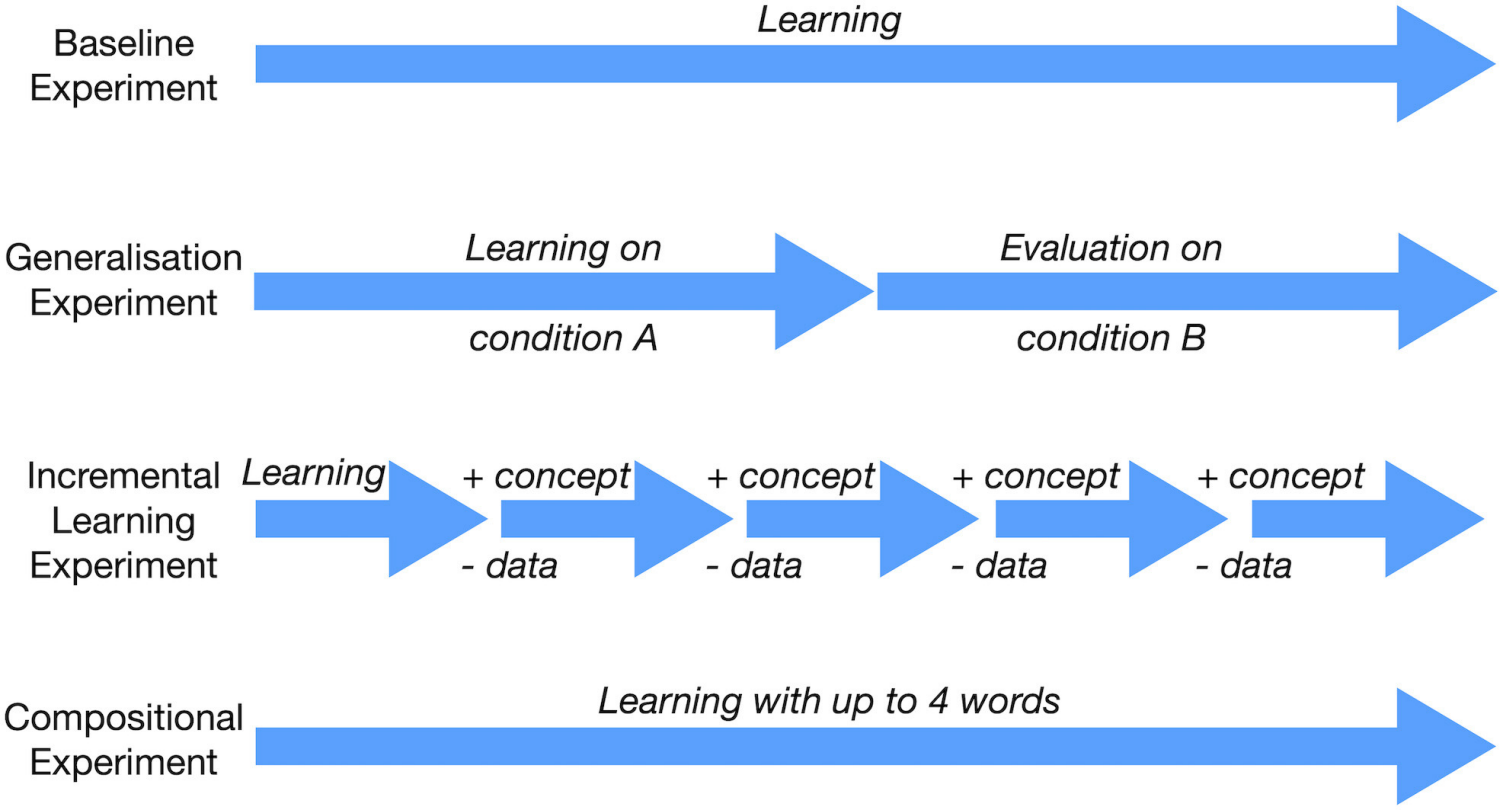
Overview of the experiments, each showcasing a particular aspect of our approach to concept learning.

In this section, we describe the various experiments designed to showcase different aspects of the proposed approach to concept learning. In the first experiment, we establish the baseline performance of our approach (section 4.1). In the following experiments, we test how well the concepts generalize (section 4.2), how they can be learned incrementally (section 4.3), and how they can be combined compositionally (section 4.4). A graphical overview of the experiments is given in Figure 5.

### 4.1. Transparent, Multi-Dimensional Concepts

In the first experiment, we validate the learning mechanisms through the language game setup laid out in section 3.1. We compare the learner's performance both using simulated (section 3.2.2) and more realistic (section 3.2.3) continuous-valued attributes. In both cases, we use scenes from the validation split of the CLEVR dataset. The learner agent is evaluated in terms of communicative success and concept repertoire size. Our goal is to validate whether or not the agent can successfully acquire and use the concepts known by the tutor. Additionally, we examine the acquired concepts to see if the agent finds combinations of attributes that are relevant in the present environment.

### 4.2. Generalization

Using the CLEVR CoGenT dataset (Johnson et al., 2017), we test if the acquired concepts are general enough to extend to unseen instances and combinations of attributes. The CLEVR CoGenT dataset consists of two conditions. In condition A, cubes can be gray, blue, brown, or yellow, cylinders are red, green, purple, or cyan and spheres can have any of these colors. In condition B, the color options for cubes and cylinders are swapped. Like the original CLEVR dataset, the CoGenT data comes with a symbolic annotation that can be transformed into continuous-valued attributes using the methods described in section 3.2. Our goal is to validate if the learner agent truly learns the concepts, independently from the statistical distribution or co-occurrences in the environment. We evaluate this by playing a number of interactions in condition A, after which we switch off learning, followed by a number of games in condition B to evaluate the communicative success. Here, we expect to see that the communicative success remains stable between condition A and B, indicating that the concepts acquired by the agent do not rely on co-occurrences in the environment, as is often the case for other types of models. Additionally, by varying the number of interactions in condition A, we gain insight into how quickly the learner can acquire concepts that are functional in the world.

### 4.3. Incremental Learning

By incrementally expanding the environment, we demonstrate the adaptivity and open-endedness of our concept learning approach. For this experiment, we created our own variation on the CLEVR dataset consisting of five splits. In each split, more concepts are added and less data is available. In the first split, we offer 10,000 images where all objects are large, rubber cubes in four different colors. In the second split, there are 8,000 images and these cubes can be large or small. Spheres and cylinders are added in the third split and the data is reduced to 4,000 scenes. The fourth split again halves the amount of data and metal objects are added. Finally, in the fifth split, four more colors are added and only 1,000 scenes are available. The splits are summarized in Table S1.

The learner agent is exposed to each of the splits consecutively, without resetting its repertoire of concepts or switching off the learning operators. We monitor the communicative success and the concept repertoire size throughout the entire experiment. Our goal in this experiment is two-fold. First, we show that the learning mechanisms can easily and quickly adjust to a changing environment. There is no need to fully or even partially re-train the repertoire when new concepts become available, nor to specify the number of concepts that are to be learned in advance, as would be the case for other types of models. By looking at the evolution of the concepts, we can study how certain attributes might become more or less important as the environment changes. Second, we again show the data efficiency of our approach by reducing the available number of scenes throughout the splits.

### 4.4. Compositional Concepts

**Figure 6:**
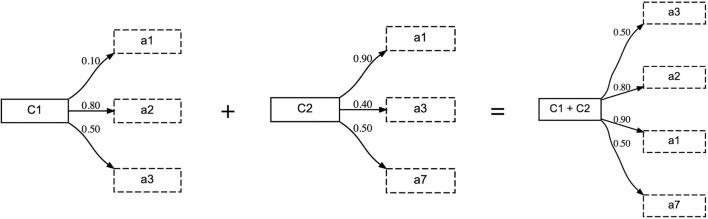
When combining concepts compositionally, the same attribute can occur multiple times. In this case, the resulting concept takes the one with the highest certainty score.

The concept representation, as described in section 3.3, can be easily extended to compositional, multi-word utterances. In order to do so, the weighted set representation of multiple concepts needs to be combined. This is achieved by an operation similar to the union operator from fuzzy-set theory (Zadeh, 1965). Given two concepts, *C*_1_ and *C*_2_, their corresponding sets of attributes are combined such that for each attribute that occurs in both concepts, the one with the highest certainty score is chosen. This is illustrated in Figure 6.

In this experiment, the tutor can use up to four words to describe the topic object. When all words in the utterance are unknown to the learner, it adopts all of them with the topic object being the initial seed. If all words are known, the learner performs the alignment using the composed concept. Due to this, not all attributes of all involved concepts will receive an updated prototypical value and certainty score, but only those that occur in the combined concept. For example, in the combined concept “C1+C2” from Figure 6, attributes “a-2” and “a-3” from concept “C1” and attributes “a-1” and “a-7” from concept “C2” will receive an update. Finally, if some words of the utterance are known and others are unknown, the learner will first adopt the unknown words and then perform alignment using the known words. In this experiment, we investigate how the communicative success, the learning speed and the resulting concepts of the agent are affected in the multi-word utterance setting and compare this to the single-word experiment described in section 4.1.

## 5. Results

In this section, we elaborate on the results of the experiments described above. In order to produce the plots, we ran all experiments five times for 10,000 interactions and averaged the results. The error bars show the standard deviation. The plots were created using a sliding window of 250 interactions. All experiments were run on the validation split of the CLEVR dataset (15K scenes), using a randomly sampled scene for every interaction. The experiments were implemented using the open-source Babel toolkit (Loetzsch et al., 2008; Nevens et al., 2019).

### 5.1. Transparent, Multi-Dimensional Concepts

**Figure 7:**
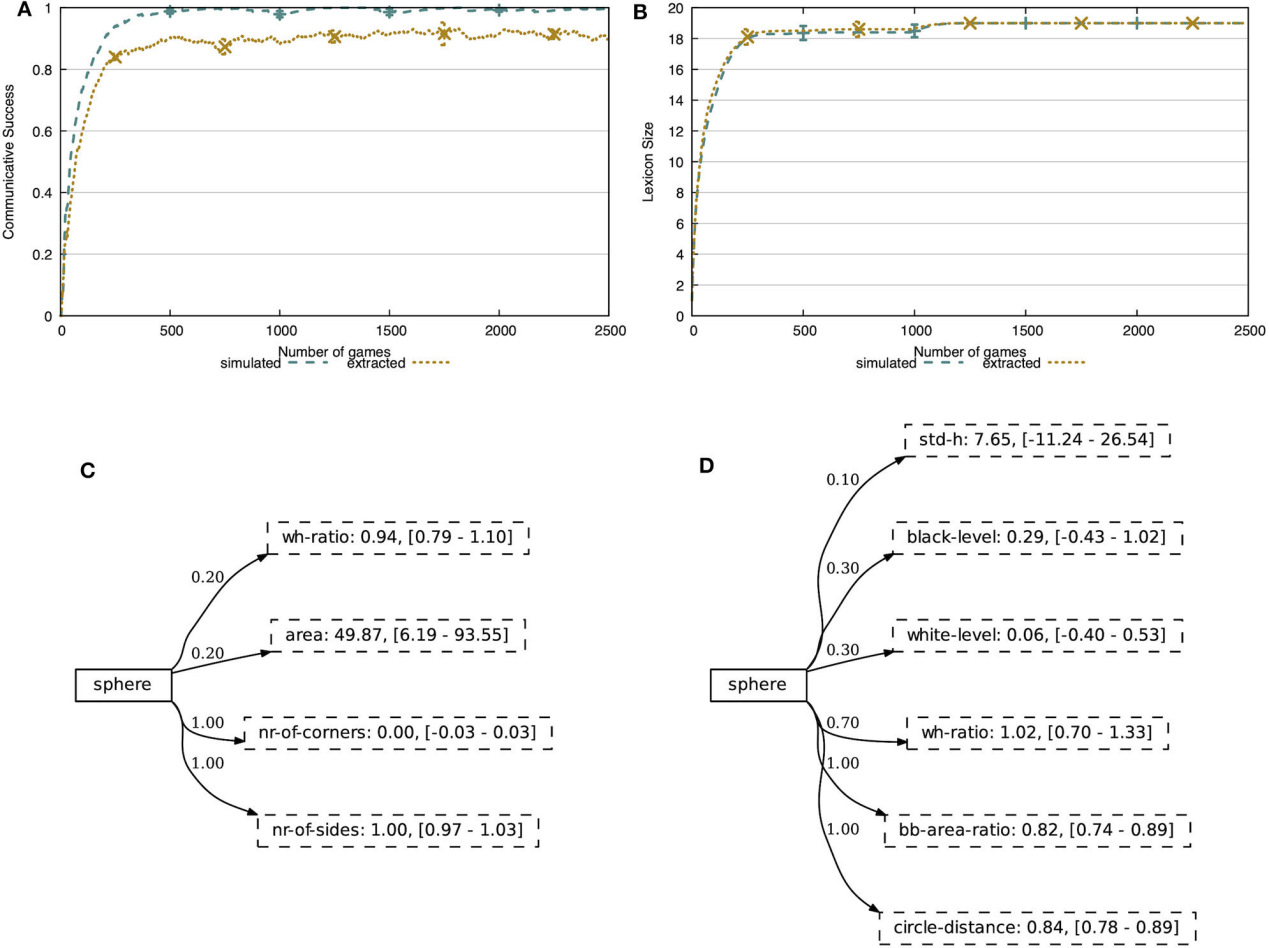
**(A)** The communicative success rises quickly and achieves 100% in the simulated world and 91% in the noisy world. **(B)** In both environments, the agent acquires exactly 19 concepts. The concepts are human-interpretable and capture discriminative combinations of attributes. The concept sphere focusses on attributes related to shape, both in the simulated environment **(C)** and the extracted environment **(D)**. Attributes with certainty score 0 are hidden.

In the first experiment, we validate the learning mechanisms proposed earlier in this paper. We evaluate the learner agent on its ability to successfully communicate and on its repertoire of concepts, both in the more simple, simulated environment and in the more realistic, noisy environment. In Figure 7A, we show the communicative success of the agents in these environments. The agents are able to achieve 100% communicative success in the simulated world, after merely ~500 interactions. From the same figure, we see that the learning mechanisms perform somewhat less good in the more realistic, noisy environment. The agents achieve a fairly stable level of communicative success after ~500 interactions, reaching 91% communicative success (0.3% standard deviation).

Figure 7B shows the lexicon size of the learner agent in both environments. Just like the communicative success, we see that it quickly increases and stabilizes at 19 concepts, which are all concepts present in the CLEVR dataset. We cut off these figures after 2,500 of the 10,000 interactions, since the metrics reached a stable level.

The concept representation proposed in this work allows for a clear and easy to interpret view on the learned concepts. We demonstrate this in Figures 7C,D, showing the concept sphere obtained after 5,000 interactions in both the simulated and noisy environments. In both cases, we see that a few attributes have become important for the learner, reflected by the high certainty scores. In the simulated world, these are *nr-of-corners* and *nr-of-sides*, while in the noisy world these are the *width-height ratio*, the *circle-distance* and *bb-area-ratio*. The *circle-distance* attribute represents the Hamming distance between the contour of the object and the minimal enclosing circle and the *bb-area-ratio* attribute represents the ratio between the area of the object and the area of its bounding box. All of these attributes are indeed intuitively shape-related. We give an overview of all learned concepts obtained in the simulated world and the noisy world in Figures S1, S2, respectively.

With this experiment, we have shown that the learner agent can automatically distill meaningful concepts from a stream of continuous data, in the form of discriminative subsets of attributes and their prototypical values, and is able to successfully use them in communication. Furthermore, as these concepts are expressed using human-interpretable feature channels, the model and resulting repertoire of concepts is completely transparant.

### 5.2. Generalization

In the generalization experiment, we show that the agent's ability to learn the concepts is completely independent from the statistical distributions or co-occurrences in the dataset. For this experiment, we use the CLEVR CoGenT dataset, which consists of two conditions. The agent first learns during a number of interactions in condition A. Afterwards, learning operators are turned off and we evaluate the communicative success of the agent in condition B for the remainder of the interactions. We expect the agents to remain at a stable level of communicative success when making the transition from condition A to B. We again evaluate on both the simulated environment and the noisy environment. Additionally, we vary the amount of training interactions on condition A to test the speed at which the learner agent can acquire useable concepts.

**Figure 8:**
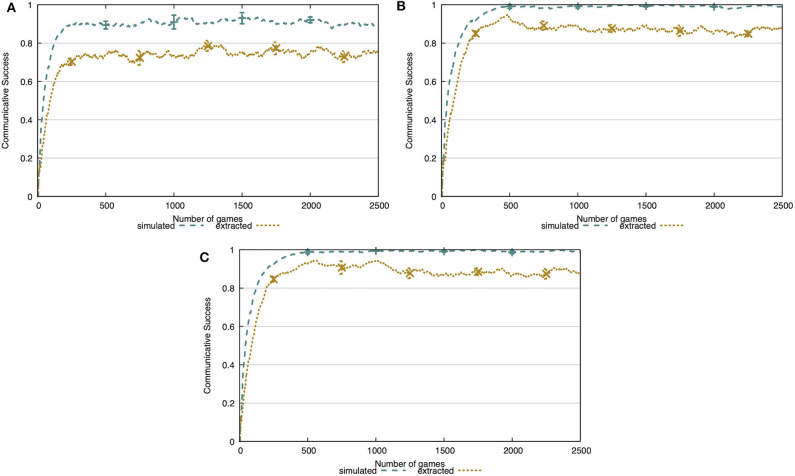
Communicative success after learning for 100 interactions **(A)**, 500 interactions **(B)**, or 1,000 interactions **(C)** in condition A. The concepts are learned completely independently from the co-occurrences in the environment. The agents achieve the same level of communicative success as in the previous experiment, given at least 500 interactions in condition A.

In Figure 8, we show the communicative success of the agents both during learning in condition A and evaluation in condition B. From this figure, it is clear that the learner agent cannot reach the same level of success as the previous experiment after 100 training interactions. However, with only 500 training interactions this level of success is achieved. This indicates that the learner's repertoire of concepts is shaped quickly and is sufficient to have successful interactions. Additionally, when transitioning from condition A to B, there is no decrease in communicative success in the simulated environment and only a minor decrease in the noisy environment. This indicates that the concepts acquired by the agent abstract away over the observed instances.

**Figure 9:**
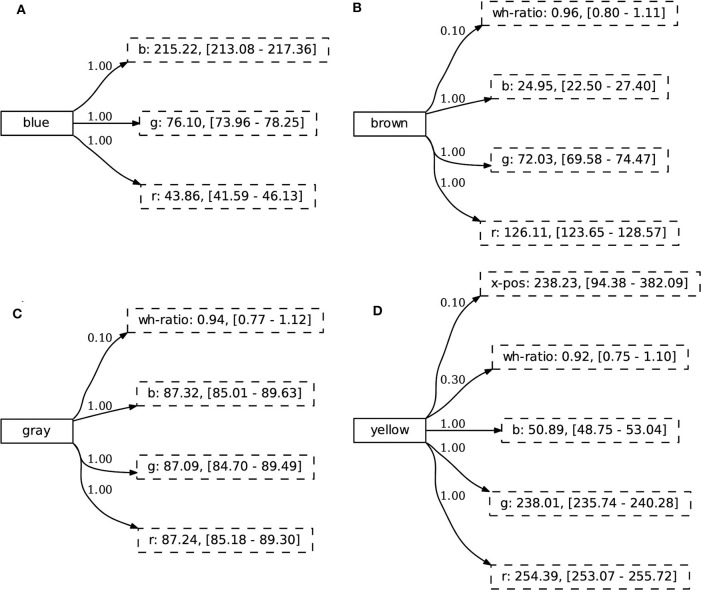
A subset of the agent's repertoire of concepts after the generalization experiment. In condition A, the concepts blue **(A)**, brown **(B)**, gray **(C)**, and yellow **(D)** are always observed as cubes or spheres. The agent is not “distracted” by statistical distributions of the environment and learns combinations of attributes that are relevant to solve the communicative task.

To further investigate the generalization abilities of the learner, we study the acquired concepts. Remember that in condition A in the CoGenT dataset, cubes can be gray, blue, brown, or yellow, cylinders have a set of different colors and spheres can be any color. In Figure 9, we study the concept representation of the colors for cubes after being learned on condition A for 500 interactions. If the agent would rely on co-occurrences of the dataset, the concept representation of these colors could contain attributes related to shape, since each time one of these colors occurs it is either a cube or a sphere. Additionally, the cube and sphere have the same value for the *wh-ratio* attribute, so it could be considered discriminative in some cases. From Figure 9, we see that even though this feature is present in some of the concepts, its certainty score is very low. Hence, the agent does not focus on particular dataset co-occurrences and is able to generalize over various observations. We attribute this to the notion of discrimination, which will make sure that only relevant attributes obtain a high certainty score.

### 5.3. Incremental Learning

**Figure 10:**
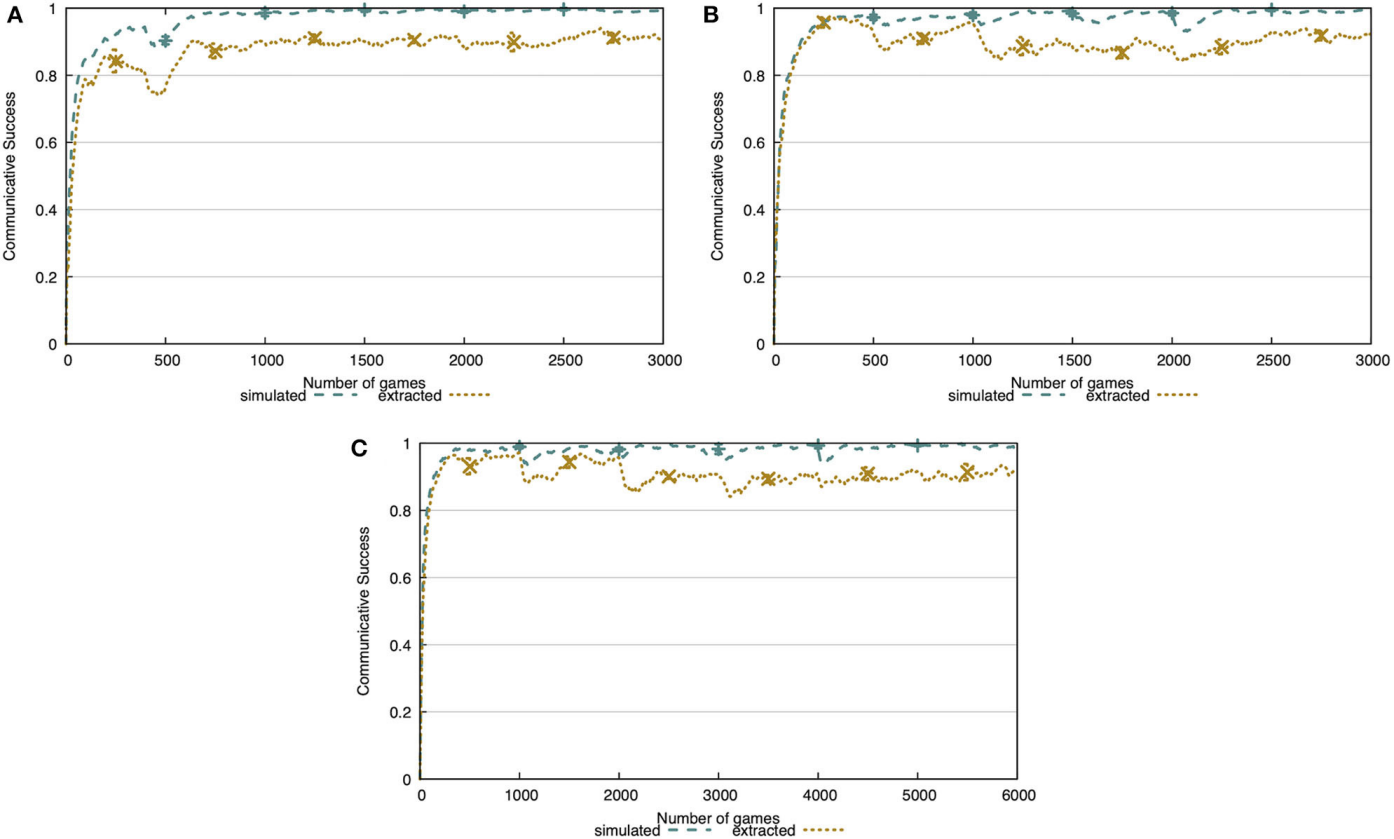
Communicative success in the incremental learning experiment. A new split is introduced every 100 interactions **(A)**, 500 interactions **(B)**, or 1,000 interactions **(C)**. The learning mechanism is completely open-ended, allowing the agent to adapt to a changing environment without any issues. Note that the x-axes vary to best show the changes in communicative success.

Our approach to concept learning is completely open-ended and has no problems dealing with a changing environment. We validate this through an incremental learning experiment where, over the course of 10,000 interactions, the number of available concepts increases. We vary the amount of interactions before new concepts are introduced between 100, 500, and 1,000 interactions. The learning mechanisms are able to adjust almost instantly to these changes, as is shown in Figure 10. In the simulated world, we see minor drops in communicative success when transitioning from one phase to the next. These are more present in the noisy world, but the agent quickly recovers from it.

**Figure 11:**
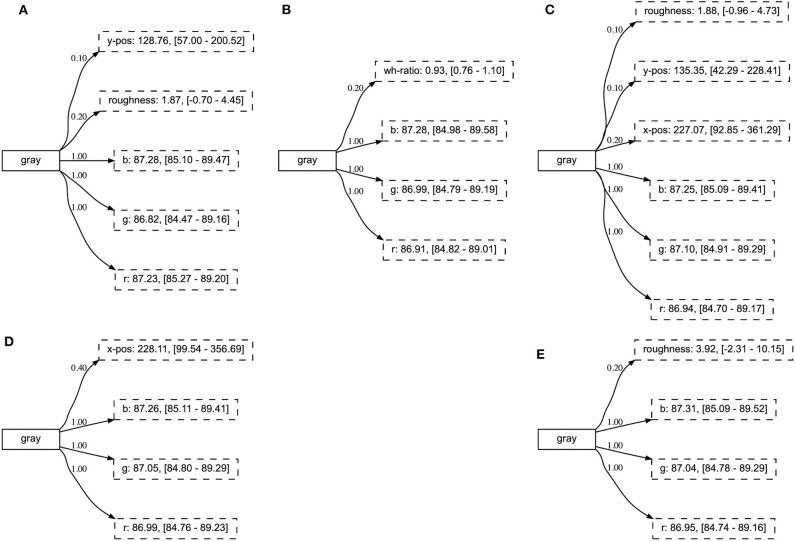
The concept gray after each of the five phases: **(A)** phase 1, **(B)** phase 2, **(C)** phase 3, **(D)** phase 4, and **(E)** phase 5. The relevant attributes obtain a high certainty score after the first phase of the experiment.

**Figure 12:**
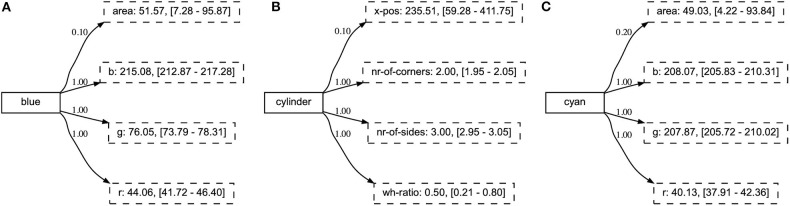
The final representation of concepts introduced in various phases of the experiment. The concept blue was introduced in phase 1 **(A)**, cylinder in phase 3 **(B)**, and cyan in phase 5 **(C)**.

If we investigate the concepts in the incremental learning experiment, we find that the relevant attributes have obtained a high certainty score already after the first phase of the experiment (see Figure 11). Consequently, these remain stable over the various phases, while other attributes never achieve high certainty scores. Additionally, we note that the resulting concepts have the same high-scoring attributes as those obtained in the baseline experiment, independent of the phase in which they were introduced (see Figure 12).

### 5.4. Compositional Utterances

**Figure 13:**
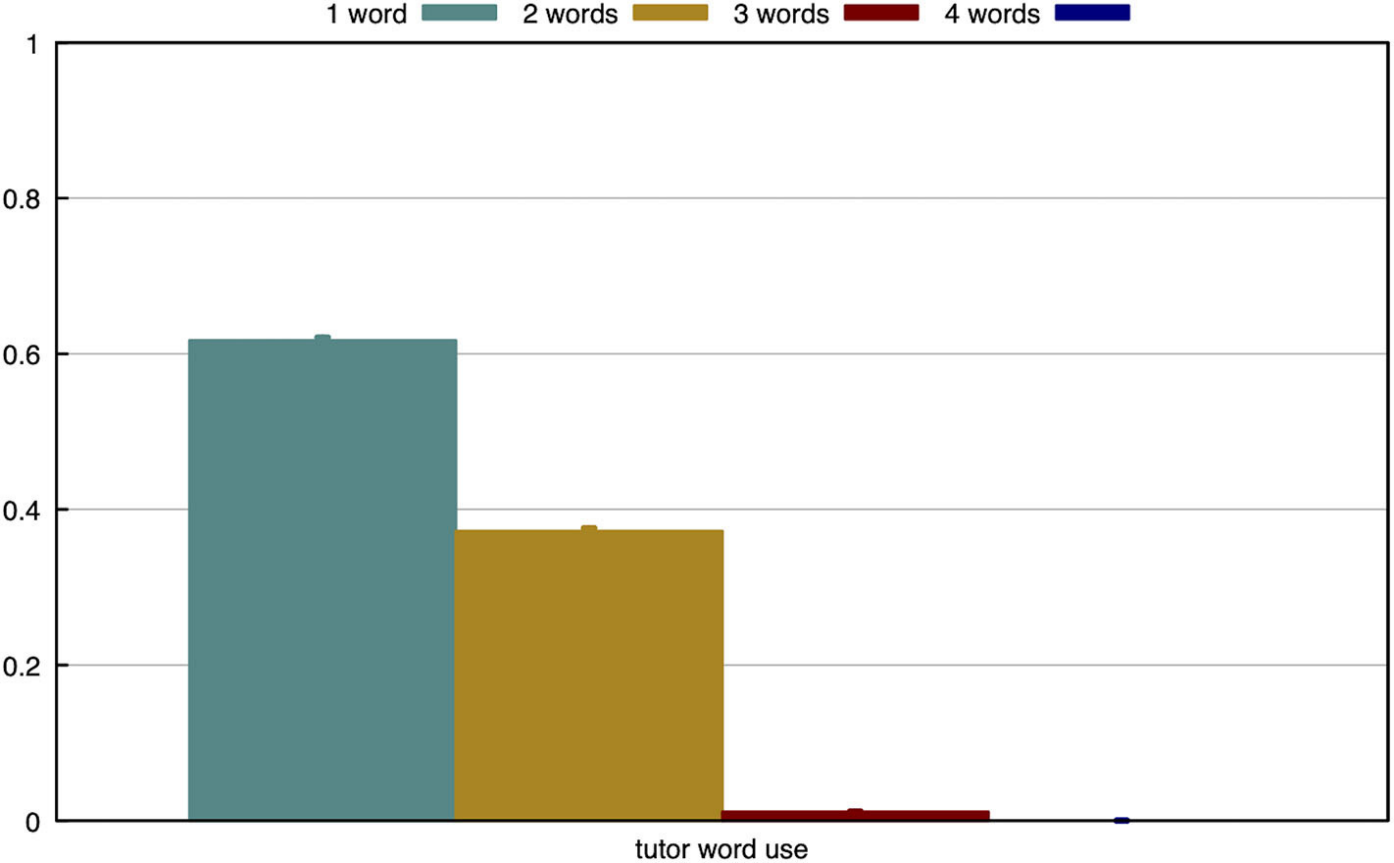
The tutor describes 63% of the objects with a single word, 36% of the objects with two words and 1% with three words.

In the final experiment, we find that the agent is successful at learning the separate concepts, even if they are combined in compositional utterances. To test this, we allow the tutor to use up to four words when describing an object. It is important to note that the tutor will always generate the shortest discriminative utterance, as described in section 3.4. In Figure 13, we measure how often the tutor uses different utterance lengths. From this, it is clear that most objects can be described using a single word. Slightly less than 40% of objects require two words to be discriminative and only very few objects are described with three words.

**Figure 14:**
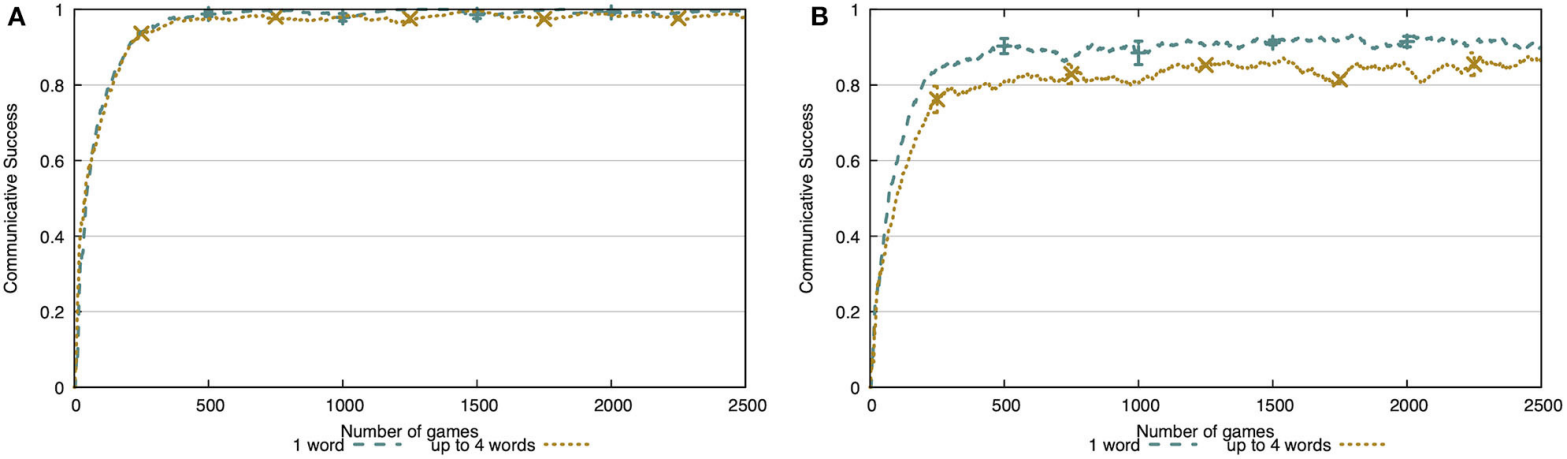
Comparison of the communicative success when the tutor uses one or up to four words. In both the simulated environment **(A)** and the extracted environment **(B)**, there is a drop in communicative success (3 and 8 p.p., respectively).

In Figure 14, we compare the communicative success when a tutor uses a single word (and skips scenes where this is not possible) and when the tutor uses up to four words. In the simulated environment (Figure 14A), communicative success drops 3 percentage points to 97%. In the noisy environment (Figure 14B), the communicative success drops 8 percentage points to 83%. With this experiment, we show that the agent is capable of extracting the discriminative attributes and their prototypical values for each concept and, at the same time, learning the meaning of each word separately in a multi-word utterance.

**Figure 15:**
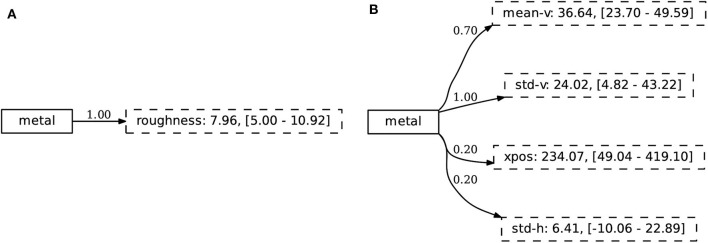
The concepts learned in the compositional experiment capture discriminative sets of attributes that are intuitively related to the concept they describe. We show the concept metal in both **(A)** the simulated and **(B)** extracted environment.

Finally, we consider the repertoire of concepts and find, similar to the first experiment, that the agent has found discriminative sets of attributes that are intuitively related to the concept they describe. The concept metal is shown in Figure 15, both for the simulated and noisy environment. Interestingly, we note from this Figure that the agent has learned to identify the material of an object through the “value” dimension of the HSV color space.

## 6. Discussion and Conclusion

In order to be able to communicate and reason about their environment, autonomous agents must be able to abstract away from low-level, sensori-motor data streams. They therefore require an abstraction layer that links sensori-motor experiences to high-level symbolic concepts that are meaningful in the environment and task at hand. A repertoire of meaningful concepts provides the necessary building blocks for achieving success in the agent's higher-level cognitive tasks, such as reasoning or action planning. Similar to how humans can grasp a concept after only a few exemplars, an autonomous agent should ideally acquire these concepts quickly and with relatively little data. Learned concepts should be general enough to extend to similar yet unseen settings. As the environment of the agent can change or new concepts can be introduced at any time, the learning methodology should also be adaptive and allow for incremental learning. Finally, to truly understand the reasoning processes of an autonomous agent, its learning mechanisms and representations should be fully transparent and interpretable in human-understandable terms.

The task of concept learning has been considered in various subfields of AI. Deep Learning approaches, for example, offer a very powerful paradigm to extract concepts from raw perceptual data, achieving impressive results but thereby sacrificing data efficiency and model transparency. Version space learning offers a more interpretable model but has difficulties in handling noisy observations. Most similar to the approach presented in this paper is work from the robotics community, considering tasks such as perceptual anchoring and affordance learning. However, these tasks focus mostly on a single robot extracting concepts from observations of the world around it. In this work, we argue for interactive learning through the language game paradigm. The notion of discrimination plays a central role in forming the concepts, thereby ensuring the generality and adaptivity of the concepts such that these are relevant in the agent's environment. Additionally, our method offers an explainable concept representation, acquired through a data efficient and incremental method. Each of these properties was highlighted in a dedicated experiment.

In sum, we have presented a novel, discrimination-based approach to learning meaningful concepts from streams of sensory data. For each concept, the agent finds discriminative attribute combinations and their prototypical values. We have shown that these concepts (i) can be acquired quickly with relatively few data points, (ii) generalize well to unseen instances, (iii) offer a transparent and human-interpretable insight in the agent's memory and processing, (iv) are adaptive to changes in the environment, and (v) can be combined compositionally. These properties make this work highly valuable for the domains of robotics and interactive task learning, where interpretability, open-endedness and adaptivity are important factors. Once a repertoire of symbolic concepts, abstracting away over the sensori-motor level, has been acquired, an autonomous agent can use it to solve higher-level reasoning tasks such as navigation, (visual) question answering, (visual) dialog and action planning.

In order to ensure that the learned concepts are human-interpretable, the methodology starts from a predefined set of human-interpretable features that are extracted from the raw images. While we argue that this is necessary to achieve true interpretability, it can also be seen as a limitation inherent to the methodology. However, this limitation cannot be lifted without losing interpretability that the method brings.

## Data Availability Statement

The datasets generated for this study are available on request to the corresponding author.

## Author Contributions

All authors contributed to the conceptual foundations and article writing. JN conducted the experiments.

## Conflict of Interest

The authors declare that the research was conducted in the absence of any commercial or financial relationships that could be construed as a potential conflict of interest.

## Footnotes

^1^This information is available at https://github.com/facebookresearch/clevr-dataset-gen.
