# Supplementary material for: From Continuous Observations to Symbolic Concepts: A Discrimination-Based Strategy for Grounded Concept Learning
Source: Front Robot AI. 2020 Jun 26;7:84. doi: 10.3389/frobt.2020.00084 (PMC7806012; doi:10.3389/frobt.2020.00084)
Supplement: Supplementary file 1 [file Data_Sheet_1.pdf]

frontiers

Supplementary Material

1 SIMULATED WORLD CONCEPTS

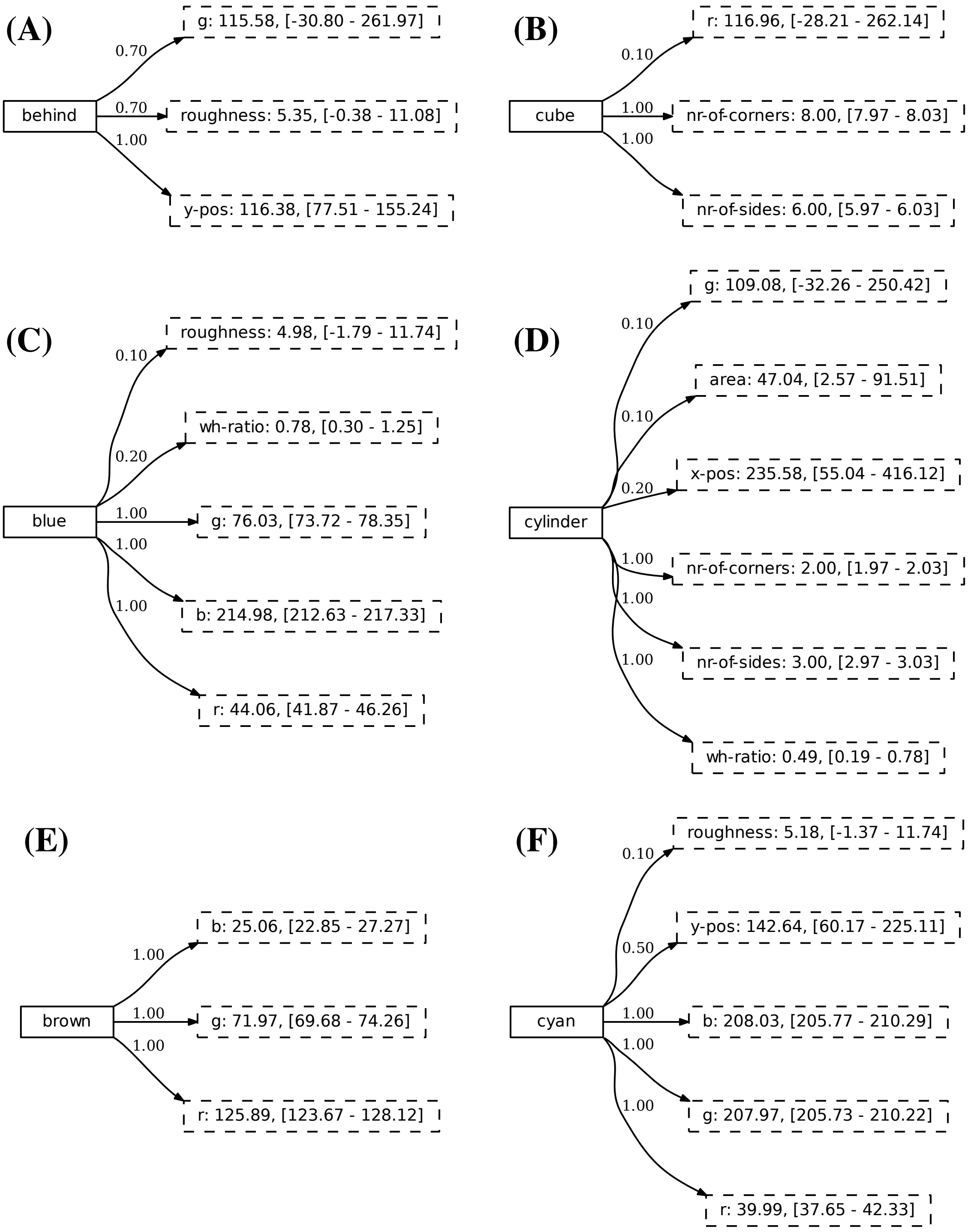

Figure S1: All concepts learned by the agent in the simulated world after 10.000 interactions

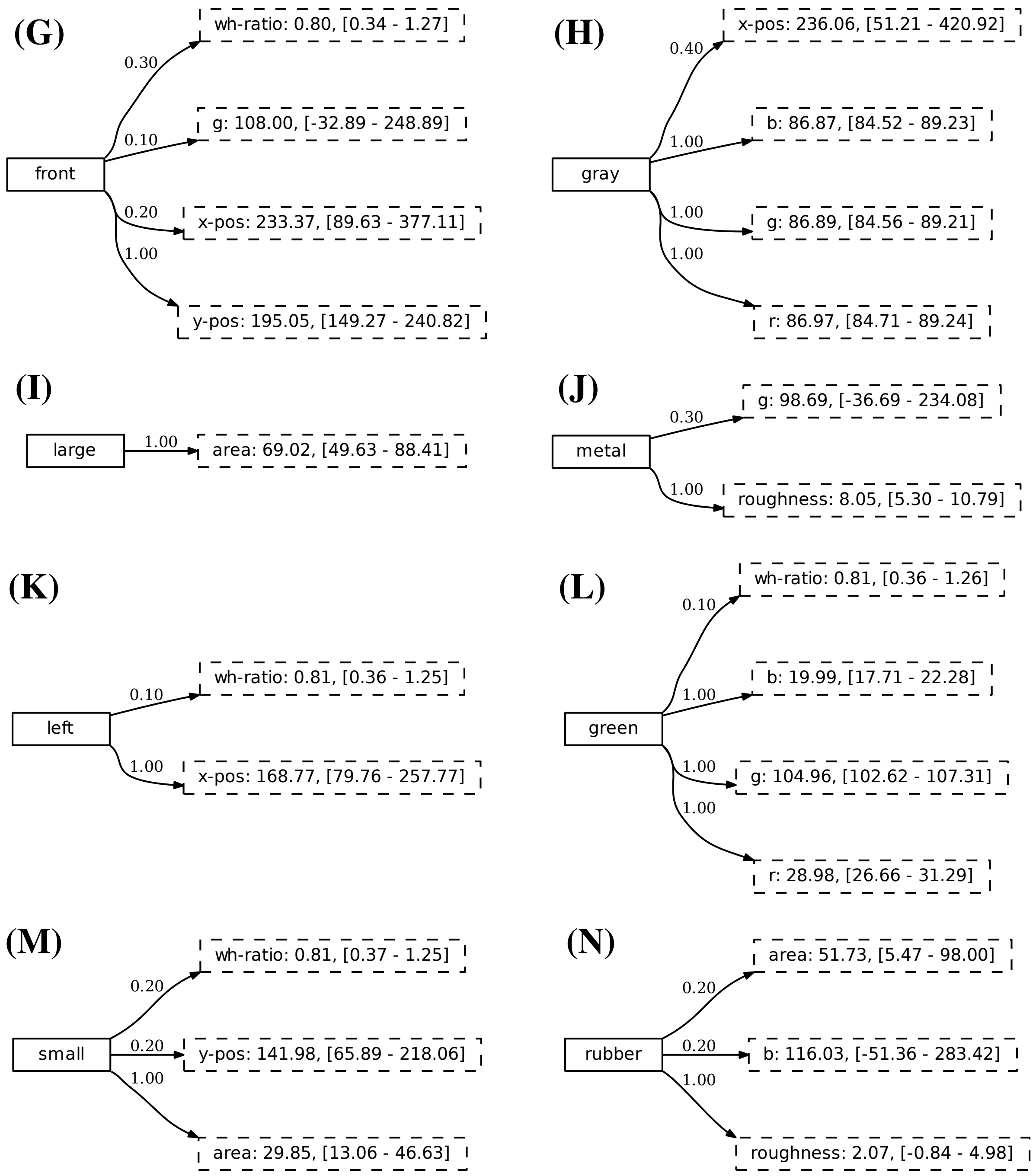

Figure S1: All concepts learned by the agent in the simulated world after 10.000 interactions

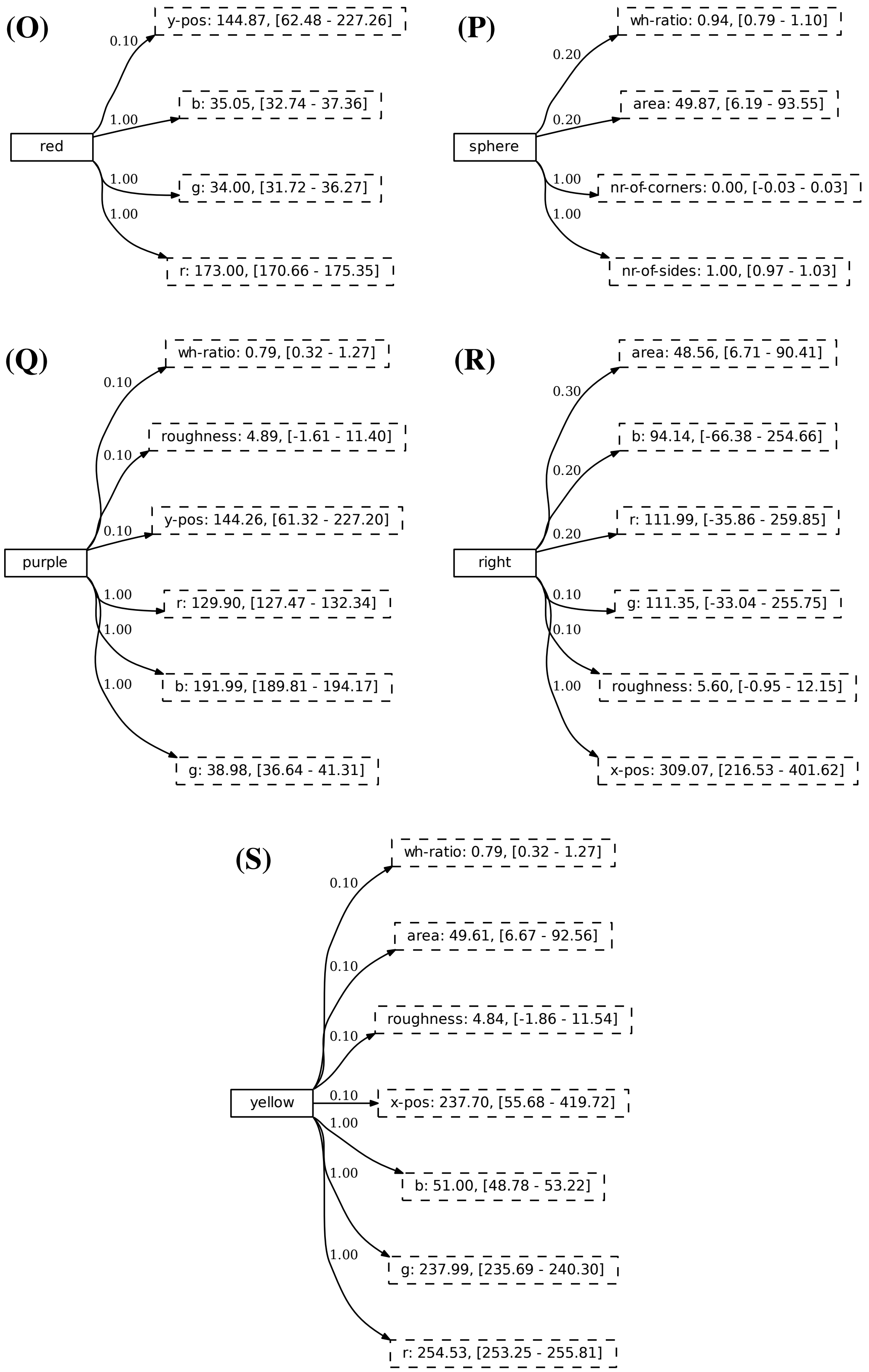

Figure S1: All concepts learned by the agent in the simulated world after 10.000 interactions

2 EXTRACTED WORLD CONCEPTS

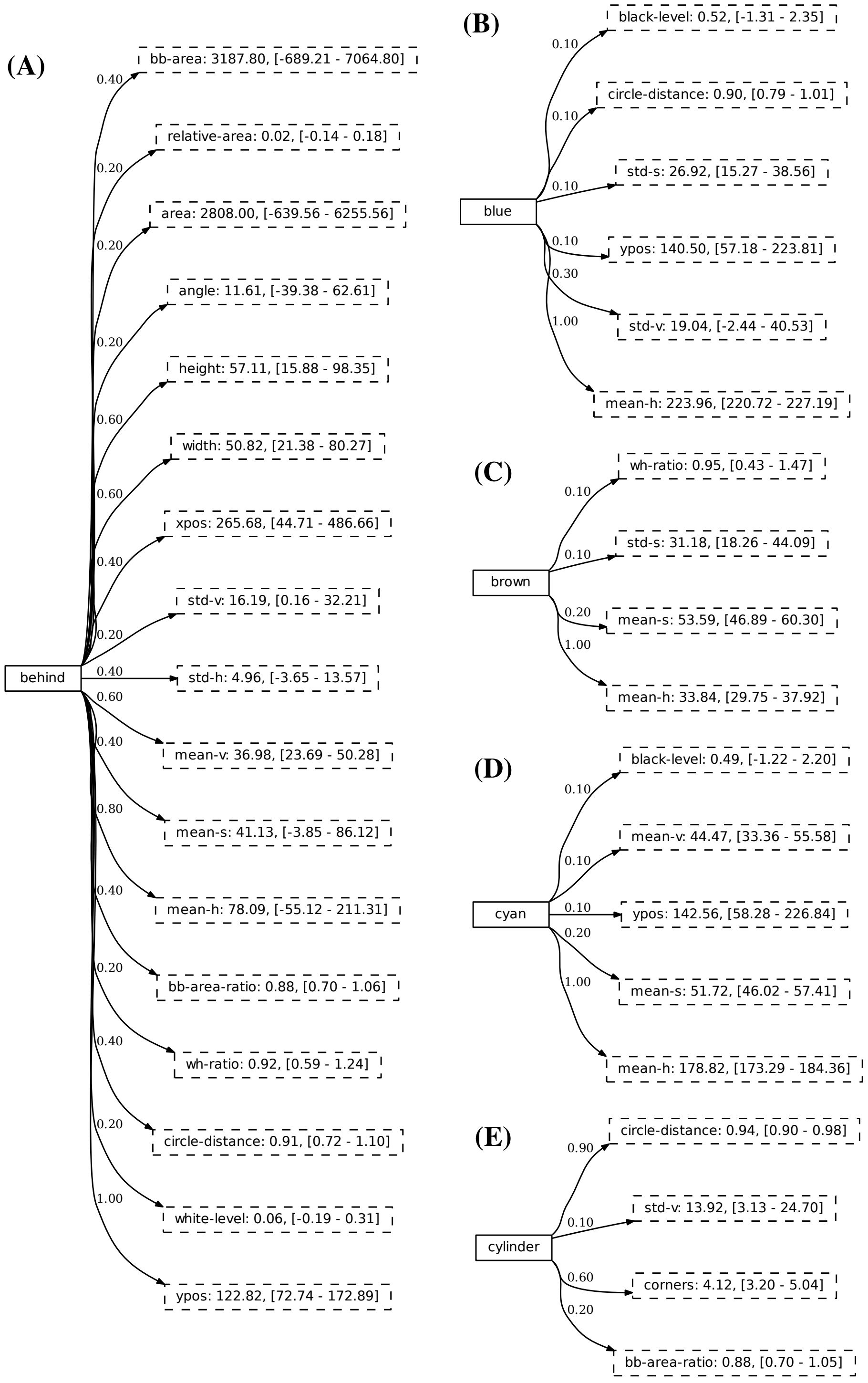

Figure S2: All concepts learned by the agent in the noisy world after 10.000 interactions

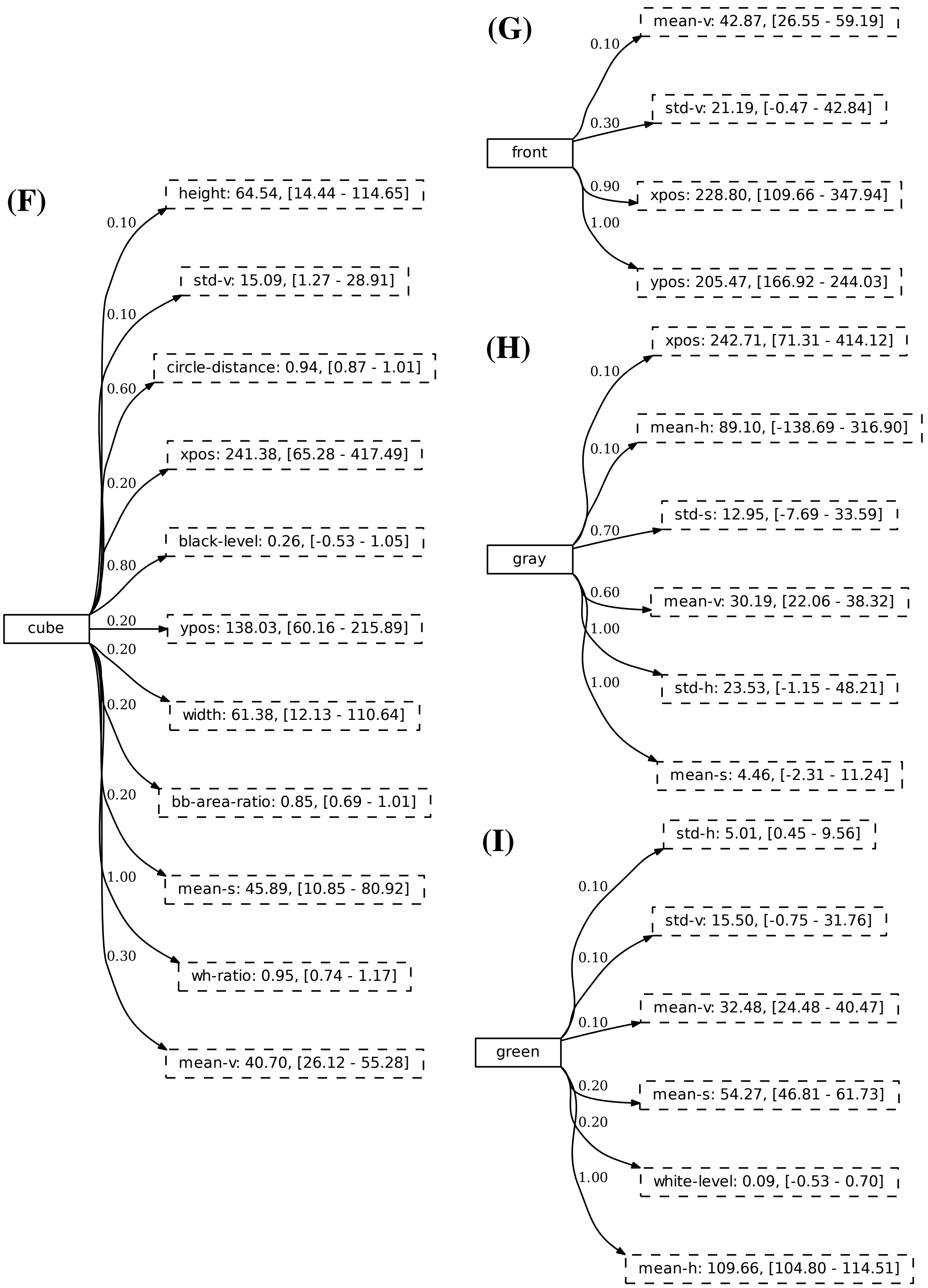

Figure S2: All concepts learned by the agent in the noisy world after 10.000 interactions

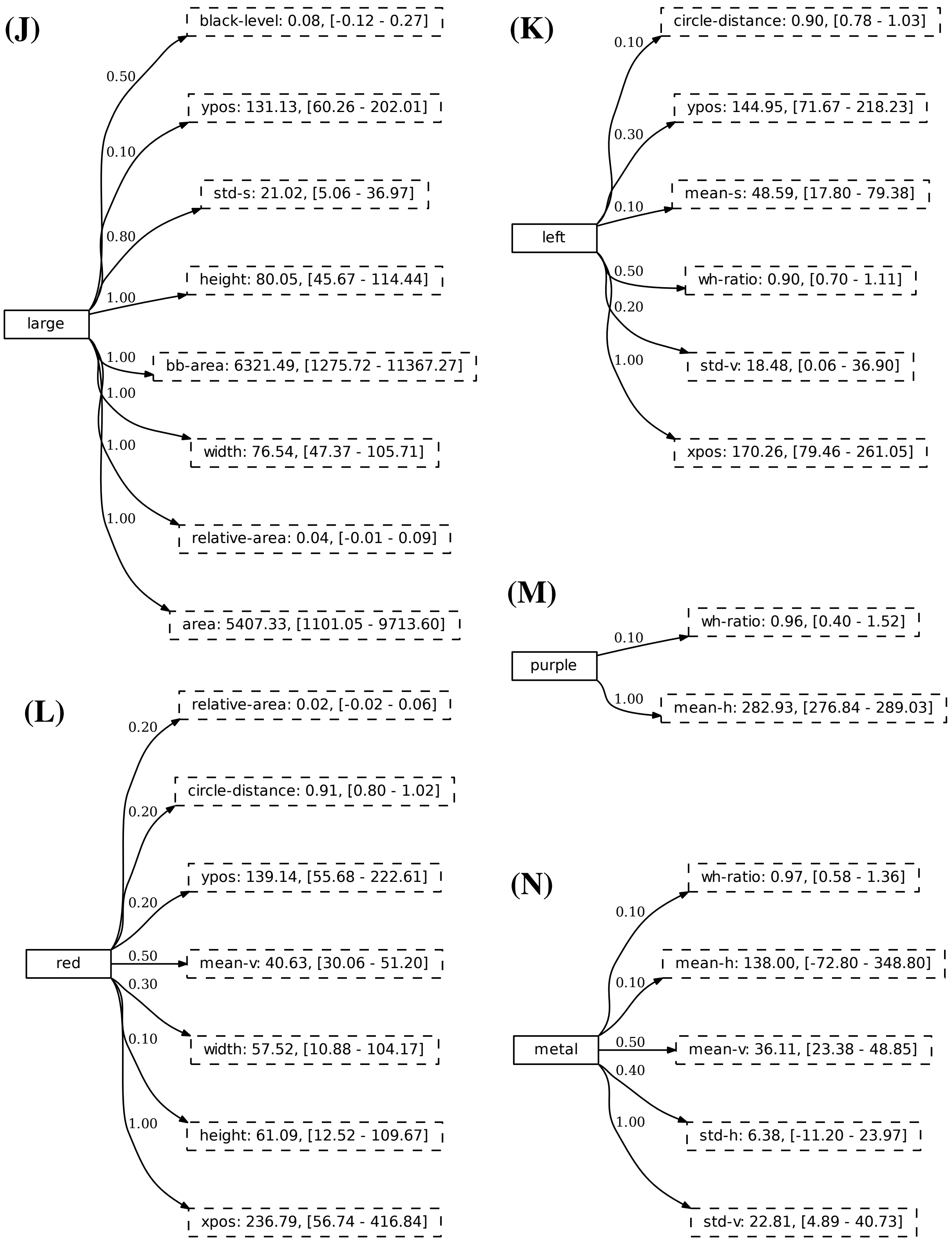

Figure S2: All concepts learned by the agent in the noisy world after 10.000 interactions

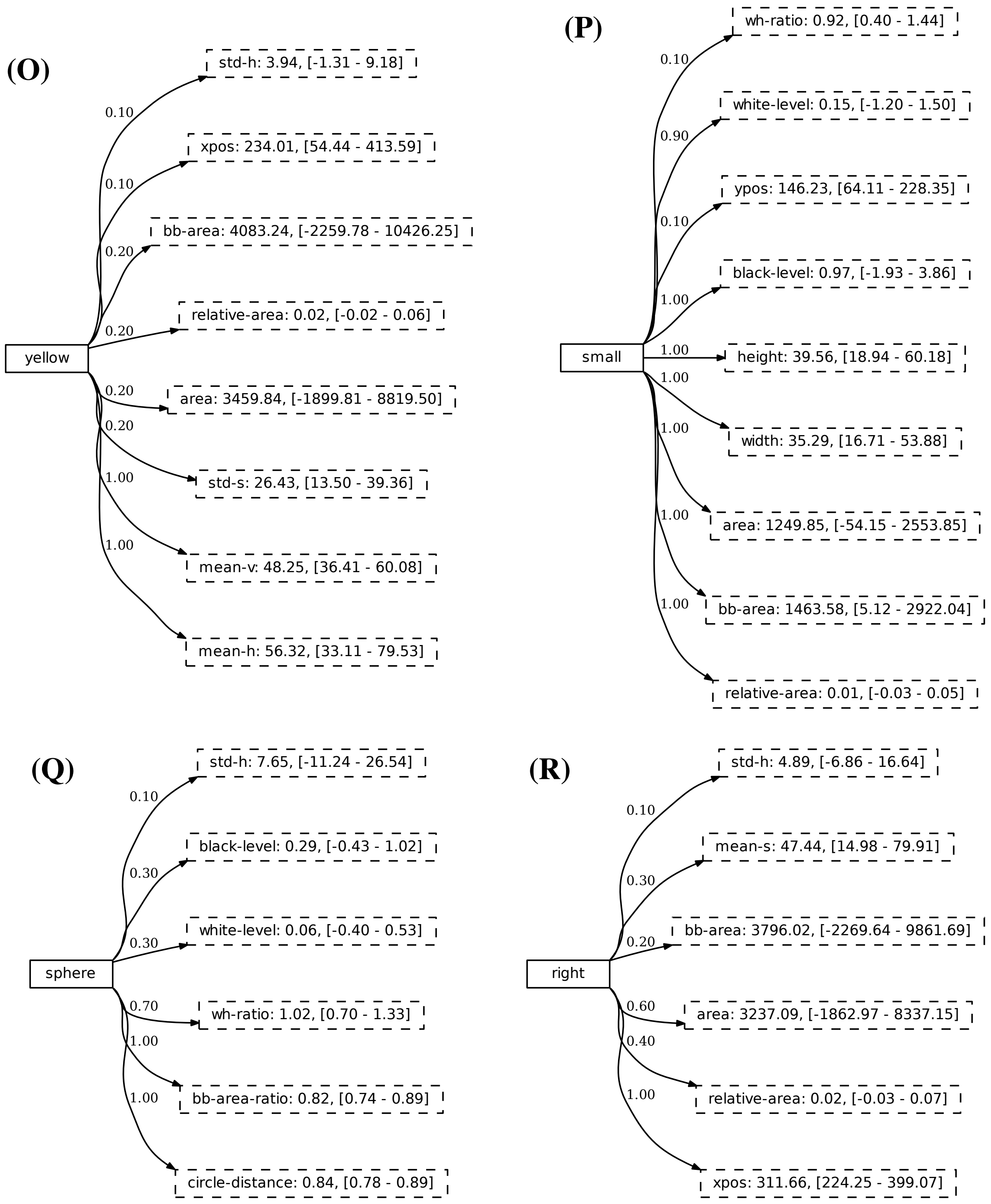

Figure S2: All concepts learned by the agent in the noisy world after 10.000 interactions

(S)

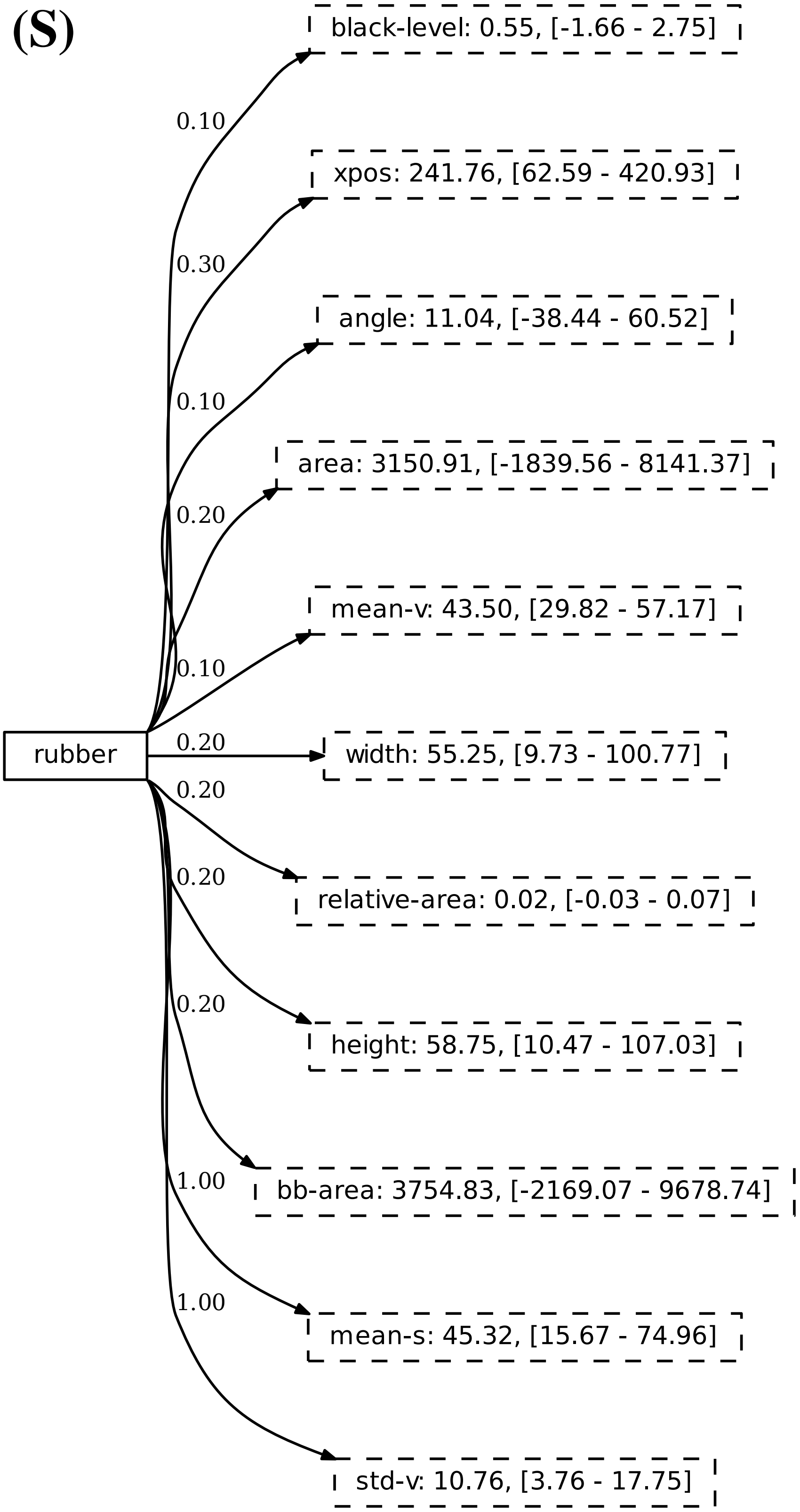

Figure S2: All concepts learned by the agent in the noisy world after 10.000 interactions

3 SUPPLEMENTARY TABLES

Split	Concepts	Scenes
1	GRAY, RED, BLUE, GREEN, CUBE, RUBBER, LARGE	10000
2	SMALL	8000
3	SPHERE, CYLINDER	4000
4	LARGE	2000
5	BROWN, PURPLE, CYAN, YELLOW	1000

Table S1. Summary of the concepts added in each split of the incremental learning dataset.
